# Trajectory-based computational analysis of the quantum–classical transition in asymmetrically coupled spin–boson models

**DOI:** 10.1016/j.csbj.2026.01.006

**Published:** 2026-01-16

**Authors:** Teerapat Uthailiang, Purin Issarakul, S. Boonchui

**Affiliations:** a Department of Physics, Faculty of Science, Kasetsart University, Bangkok, 10900, Thailand; b Special Research Incubator Unit (SRIU), Faculty of Science, Kasetsart University, Bangkok, 10900, Thailand

**Keywords:** Quantum state transition, Spin-boson model, Stochastic Langevin-Itô equation, Hierarchical equations of motion

## Abstract

Understanding how quantum coherence is regulated by structured environments is essential for elucidating energy-transfer mechanisms in photosynthetic light-harvesting complexes. In this work, we present a trajectory-based computational analysis of the quantum–classical transition in asymmetrically coupled spin–boson models, motivated by exciton–phonon interactions in the phycobiliprotein PC645 complex. The model captures site-dependent environmental coupling that mimics pigment-specific dissipation pathways in biological systems. We employ three complementary approaches: a Redfield master equation in the Bloch-vector representation, numerically exact hierarchical equations of motion (HEOM), and a stochastic Schrödinger equation that generates ensembles of quantum trajectories. Within the stochastic framework, environmental backaction is interpreted as a continuous measurement process, giving rise to a time-dependent dynamical corridor on the Bloch sphere. The corridor width provides a quantitative measure of coherence loss and defines the quantum–classical crossover time. Our results show that moderate asymmetric coupling can sustain coherence and enhance directional population transfer, whereas strong coupling rapidly suppresses quantum trajectories. These findings offer mechanistic insight into environmentally assisted energy transfer and coherence regulation in photosynthetic pigment–protein complexes.

## Graphical abstract

**Figure:**
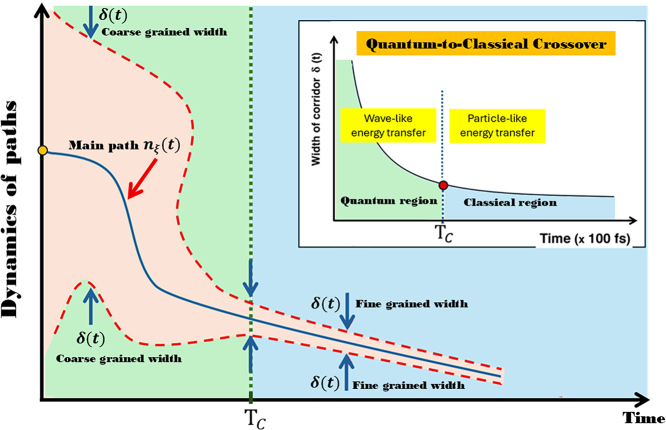

## 1 Introduction

Quantum effects in biological systems and condensed-phase chemical processes have attracted sustained interest over the past decades, ranging from excitonic energy transfer in photosynthesis [1], [2], [3], [4] to quantum-inspired mechanisms in neural systems [5], biomolecular recognition such as SARS-CoV-2 receptor interactions [6], and cavity-modified reaction dynamics in condensed phases [7]. A common theoretical viewpoint is that these phenomena are most naturally described within the framework of *open quantum systems*, where a reduced quantum subsystem interacts with a complex environment, leading to dissipation, decoherence, and non-Markovian memory effects.

Among the paradigmatic models of open-system dynamics, the spin–boson model plays a central role. It describes a two-level subsystem (spin or qubit) coupled to a bosonic (or fermionic) bath [8], [9], [10]. This model has been extensively studied using both analytical and numerical approaches, including direct integration schemes [11], [12], [13], the hierarchical equations of motion (HEOM) [14], [15], [16], and the time-evolving density-operator with orthogonal polynomials algorithm (TEDOPA) [17]. Importantly, these methods have also enabled quantitative modelling of biologically relevant light-harvesting assemblies, for instance excitonic energy transfer in the Fenna–Matthews–Olson (FMO) complex using TEDOPA [18] and HEOM [19].

In open quantum systems, the system–bath coupling is the primary mechanism responsible for decoherence and relaxation, and thus it decisively shapes quantum-state transitions and energy-transfer dynamics. Prior studies have shown that the coupling strength and bath structure can govern transfer efficiency and coherence lifetimes in photosynthetic complexes [4], [20]. Moreover, experiments on the PC645 complex have reported field-induced detuning of vibronic resonances, where strong magnetic fields can shift excitonic energy levels while leaving nuclear vibrational modes—and hence the underlying system–bath coupling—largely unaffected [21].

Motivated by these developments, we investigate how the *choice of system–bath coupling operator* controls the interplay between coherent driving and measurement-induced decoherence in the spin–boson model. Specifically, we analyse how variations in coupling strength [22], operator-induced asymmetry (mimicking pigment-specific exciton–phonon interactions in light-harvesting complexes) [1], [2], [3], [4], [7], [23], [24], [25], [26], [27], [28], bath temperature, and a time-dependent external drive [21], [29] jointly determine the transition dynamics. We employ three complementary approaches: i) the Redfield master equation in Lindblad form as a conventional weak-coupling description [30]; ii) HEOM, developed by Tanimura and Kubo [14], [15], as a numerically exact benchmark across weak to strong coupling; and iii) a stochastic Langevin–Itô (stochastic Schrödinger) formulation [31], [32] that generates ensembles of quantum trajectories.

Beyond cross-validating these approaches, we introduce a continuous-measurement interpretation of the stochastic trajectories: environmental backaction is encoded statistically through a path-integral weighting functional, which defines a finite *dynamical corridor* of admissible paths on the Bloch manifold. This viewpoint yields an intuitive geometric mechanism for the quantum-to-classical crossover, whereby environmental monitoring progressively narrows the corridor and suppresses large-deviation trajectories.

## 2 Theory and methods

### 2.1 Model Hamiltonian

**Fig. 1:**
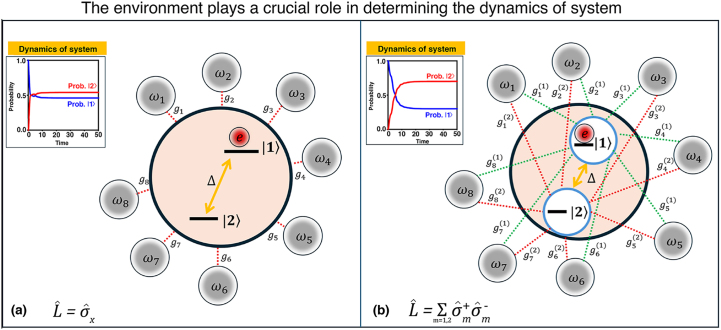
An open quantum system consists of a two-state quantum subsystem ${\hat{\text{H}}}_{\text{S}}$, an environment (bath), and their interaction. The interaction ${\hat{\text{H}}}_{\text{SB}}$ between the quantum subsystem and the environment plays a crucial role in determining the system’s dynamics and the noise sources that affect its performance. In the case of the system operator (a)${\hat{\sigma }}_{x}$ (transition coupling) and (b)$\sum_{m=1,2}{\hat{\sigma }}_{m}^{+}{\hat{\sigma }}_{m}^{-}$ (asymmetric local bath coupling).

The spin-boson model provides a conceptual framework for describing open quantum systems that model biological processes, where a two-level subsystem interacts with a single environmental bath to explore different system-bath coupling operators and emulate site-dependent asymmetry effects on quantum dynamics, (see Fig. 1) [8], [9], [10]. The total Hamiltonian is

(1) $$\hat{H}={\hat{H}}_{\text{S}}+\sum_{k}\hbar \omega_{k}{\hat{b}}_{k}^{†}{\hat{b}}_{k}+{\hat{H}}_{\text{SB}},$$

where ${\hat{H}}_{\text{S}}$ is the Hamiltonian of the system, ${\hat{b}}_{k}^{†}({\hat{b}}_{k})$ are the creation (annihilation) operators of the k-th vibrational mode of the bosonic bath, and ${\hat{H}}_{\text{SB}}$ the system–bath coupling Hamiltonian defined as

(2) $${\hat{H}}_{\text{SB}}=\hat{L}\sum_{k}\hbar g_{k}({\hat{b}}_{k}+{\hat{b}}_{k}^{†}),$$

with $\hat{L}$ is the system operator that couples to the bosonic bath and $g_{k}$ denotes the coupling strength between the system and the k-th vibrational mode of the bosonic bath.

In this work, we use the Hamiltonian of the system, ${\hat{H}}_{\text{S}}$, defined as given by equation [9], [10], [19],

(3) $${\hat{H}}_{\text{S}}=\frac{\hbar \epsilon }{2}{\hat{\sigma }}_{z}+\frac{\hbar \Delta }{2}{\hat{\sigma }}_{x},$$

where ϵ and Δ represent the site energy bias and the tunnelling amplitude (or the electronic coupling), respectively. We focus on investigating the effects of system-bath coupling on state transitions (or energy transfer) by modifying the system operator $\hat{L}$ as follows: i) Transition-inducing (dipole-like) coupling and ii) Asymmetric local bath coupling

(4) $$\text{i) }{\hat{L}}_{T}=c{\hat{\sigma }}_{x},\;\;\text{and}\;\;\text{ii)}\;{\hat{L}}_{AL}=\sum_{m=1,2}c_{m}{\hat{\sigma }}_{m}^{+}{\hat{\sigma }}_{m}^{-},\;\;\;\text{where}\;\;\;{\hat{\sigma }}_{1}^{\pm }={\hat{\sigma }}_{2}^{\mp },$$

where $c\;\;\text{and}\;\;c_{m}$ are the constants used to adjust the coupling strength. Two forms of coupling are considered: a transition coupling ${\hat{L}}_{T}$, which is the traditional form used to study such systems, for example, in Refs. [24], [25], [26], [27]; and an asymmetric local bath coupling ${\hat{L}}_{AL}$, which captures asymmetric interactions between sites. The local coupling is used to consider exciton-mode interactions in biological systems such as discussion of energy transfer in FMO complex [1], [2], [3], [4], [23], [28], and PC645 complex [21]. We set $g_{k}^{\left(m\right)}=c_{m}g_{k}$ where $g_{k}^{\left(1\right)}\neq g_{k}^{\left(2\right)}$ as $g_{k}^{\left(2\right)}=\text{r}g_{k}^{\left(1\right)}$ with r being the coupling strength ratio. The key findings presented here arise from our consideration that compares non-local (off-diagonal) and local asymmetric coupling operators within the same model on the role of quantum effects in biological systems, including the fundamental question of “how does the quantum-to-classical crossover occur for energy transfer in biological surroundings” which may be important in different photosynthetic systems. To choose parameter mapping to biological systems, we use these parameters (see Supplementary Note 7–7.1) that are similar to those used in simulations of photosynthetic pigment protein complexes, in particular, phycobiliproteins such as PC645 and FMO complex [1], [2], [3], [4], [21].

In addition, we examine how an external field influences state transitions within the system. This is motivated by experimental studies of perturbative or field-induced effects in various contexts—for instance, in photosynthetic processes where the application of a magnetic field has been shown to enhance energy transfer efficiency [21]. Hence, in this work, the external driving field is introduced to render the tunnelling element time-dependent as shown [29]

(5) $$\Delta (t)=\Delta +B_{0}\cos(\omega_{B}t),$$

where $B_{0}$ and $\omega_{B}$ are, respectively, the amplitude and frequency of the external (magnetic) field, and the tunnelling amplitude, Δ, is represented by the static dipole–dipole coupling, which is consistent with the molecular configuration of the biological system, and is defined as

(6) $$\Delta =\frac{1}{4\pi \epsilon_{0}}[\frac{{\vec{d}}_{1}\cdot {\vec{d}}_{2}}{|{\vec{r}}_{12}{|}^{3}}-3\frac{\left({\vec{d}}_{1}\cdot {\vec{r}}_{12})({\vec{d}}_{2}\cdot {\vec{r}}_{12}\right)}{|{\vec{r}}_{12}{|}^{5}}],$$

where ${\vec{d}}_{1(2)}$ are the dipole moments of the electronic state for the m-th state, with the separation vector ${\vec{r}}_{12}={\vec{r}}_{1}-{\vec{r}}_{2}$ and $\epsilon_{0}$ is the vacuum dielectric permittivity.

### 2.2 The quantum master equation with the Bloch vector and the HEOM method

In the study of open quantum systems, where a subsystem continuously interacts with its surrounding environment, the master equation provides the fundamental framework for describing the non-unitary evolution of the system’s density operator, $\hat{\rho }(t)$. This formalism generalises the conventional Schrödinger equation to include dissipative and decoherence effects arising from system–bath coupling, thereby capturing irreversible dynamics that cannot be represented by the unitary evolution governed solely by ${\hat{H}}_{\text{S}}$. The reduced density operator of the system is obtained by tracing out the environmental degrees of freedom, yielding an effective equation of motion for the subsystem. Under appropriate approximations, this equation assumes the Redfield form, commonly referred to as the Redfield master equation [30], [33]:

(7) $$\begin{array}{ll} \frac{d}{dt}\hat{\rho }(t) & \;=-\frac{i}{\hbar }[{\hat{H}}_{\text{S}},\hat{\rho }(t)]-\frac{1}{\hbar^{2}}\int_{0}^{t}dt^{\prime }{\text{Tr}}_{\text{B}}\{[{\hat{H}}_{\text{SB}}(t),[{\hat{H}}_{\text{SB}}(t^{\prime }),\hat{\rho }(t)]]\}\\ & \;=-\frac{i}{\hbar }[{\hat{H}}_{\text{S}},\hat{\rho }(t)]+\sum_{\mu }(\Gamma_{\mu }(\omega ,t)({\hat{L}}_{\mu }\hat{\rho }(t){\hat{L}}_{\mu }^{†}-{\hat{L}}_{\mu }^{†}{\hat{L}}_{\mu }\hat{\rho }(t))+\text{h.c.}) \end{array}$$

where the system Hamiltonian, ${\hat{H}}_{\text{S}}$, is expressed in its diagonalised form, satisfying ${\hat{\text{H}}}_{\text{S}}|\pm \rangle =\pm (\hbar \Omega /2)|\pm \rangle$ with $\Omega =\sqrt{\epsilon^{2}+\Delta^{2}}$ denotes the Bohr frequency, and ${\hat{L}}_{\mu }$ the Lindblad operator with μ=±,z that related to the eigenstate |±⟩ basis and the Bohr frequency (see the detailed calculation in Supplementary Notes 1–3). For the dissipation coefficient, $\Gamma_{\mu }(\omega ,t)$, defined following equation

(8) $$\Gamma_{\mu }(\omega ,t)=\frac{1}{\hbar^{2}}\int_{0}^{t}d\tau \;e^{i\omega \tau }C_{\mu }(\tau )=\frac{1}{2}\gamma_{\mu }(\omega ,t)+iS_{\mu }(\omega ,t),$$

and $C_{\mu }(\tau )$ is the bath correlation function can be written as

(9) $$C_{\mu }(\tau )=\int_{0}^{\infty }d\omega \;J(\omega )[\coth(\frac{\beta \hbar \omega }{2})\cos\left(\omega \tau \right)-i\sin\left(\omega \tau \right)],$$

where J(ω) is the spectral density and $\beta =1/k_{B}T$ is the inverse temperature. In this work, we focus on the following type of spectral density: the under-damped Brownian motion form [34]

(10) $$J_{U}(\omega )=\frac{\gamma \lambda^{2}\omega }{{\left(\omega^{2}-\omega_{0}^{2}\right)}^{2}+\gamma^{2}\omega^{2}}.$$

This spectral density is characterised by a resonance frequency $\omega_{0}$, a width γ, and a system-bath coupling strength λ, which is associated with $g_{k}$ for the system operator ${\hat{\sigma }}_{x}$ and $g_{k}^{\left(1\right)}\sqrt{\left|1-\text{r}\right|}$ for the system operator ${\hat{L}}_{AL}$. However, the coupling regime for both system operators is characterised by the ratio between the renormalisation energy $E_{\text{ren}}$ and the Bohr frequency Ω [35], where $E_{\text{ren}}$ is defined as (see detail in Supplementary Note 7–7.4)

(11) $$E_{\text{ren}}=\int_{0}^{\infty }d\omega \;\frac{J_{U}(\omega )}{\omega }=\frac{\pi \lambda^{2}}{2\omega_{0}^{2}}.$$

Accordingly, the weak-coupling regime corresponds to $E_{\text{ren}}/\Omega \ll 1$, the strong-coupling regime to $E_{\text{ren}}/\Omega ≳1$, and the intermediate regime typically lies within $E_{\text{ren}}/\Omega \approx 0.1$–0.5.

To solve Eq. (7) for a two-level system, we represent any operator (not necessarily Hermitian, nor unitary) as a linear combination of the identity operator and the Pauli operator vector $\hat{\vec{\sigma }}$. It is convenient to introduce the following identity [36]:

(12) $$\hat{\rho }(t)=\frac{1}{2}[\hat{I}+{\vec{n}}^{\left(av\right)}(t)\cdot \hat{\vec{\sigma }}],\;\;\;\text{and}\;\;\;{\vec{n}}^{\left(av\right)}(t)=\text{Tr}[\hat{\vec{\sigma }}\hat{\rho }(t)],$$

(13) $${\hat{H}}_{\text{S}}=H_{\text{S}}^{0}\hat{I}+{\vec{H}}_{\text{S}}\cdot \hat{\vec{\sigma }},\;\;\text{and}\;\;{\hat{L}}_{\mu }=L_{\mu }^{0}\hat{I}+{\vec{L}}_{\mu }\cdot \hat{\vec{\sigma }},$$

Here ${\vec{n}}^{\left(av\right)}(t)$ is an *ensemble-averaged* Bloch vector that generally satisfies $|{\vec{n}}^{\left(av\right)}(t)|\leq 1$ due to loss of purity, and therefore the constrained trajectories ${\vec{n}}^{\left(av\right)}(t)$ are not restricted to lie on the unit sphere. This is consistent with the physical reduction of purity induced by system–bath interactions.

By substituting (12), (13) into Eq. (7) and performing straightforward algebraic manipulation, the evolution of the corridor path, referred to as *the equation of motion* of the trajectory, is obtained as follows [4]:

(14) $$\frac{d}{dt}{\vec{n}}^{\left(av\right)}(t)=\vec{B}(t)\times {\vec{n}}^{\left(av\right)}(t)+\overset{↔}{\eta }(t)\cdot {\vec{n}}^{\left(av\right)}(t)+\vec{T}(t),$$

where

(15) $$\vec{B}(t)=\frac{2}{\hbar }{\vec{H}}_{\text{S}}-2i\sum_{\mu }(\Gamma_{\mu }(\omega ,t)L_{\mu }^{0}{\vec{L}}_{\mu }^{†}-\Gamma_{\mu }^{*}(\omega ,t)L_{\mu }^{0†}{\vec{L}}_{\mu }),$$

(16) $$\begin{array}{ll} \overset{↔}{\eta }(t)\cdot {\vec{n}}^{\left(av\right)}(t) & \;=2\sum_{\mu }(\Gamma_{\mu }(\omega ,t){\vec{L}}_{\mu }^{†}\times ({\vec{L}}_{\mu }\times {\vec{n}}^{\left(av\right)}(t))\\ & \;+\Gamma_{\mu }^{*}(\omega ,t){\vec{L}}_{\mu }\times ({\vec{L}}_{\mu }^{†}\times {\vec{n}}^{\left(av\right)}(t))), \end{array}$$

and

(17) $$\vec{T}(t)=2i\sum_{\mu }\gamma_{\mu }(\omega ,t)({\vec{L}}_{\mu }\times {\vec{L}}_{\mu }^{†}).$$

(15), (16), (17) correspond to the precession, relaxation, and torque, respectively (equation for use, see Supplementary Note 4).

For the numerical analysis, the HEOM is employed. This formalism, originating from the Feynman–Vernon influence functional approach [15], [19], utilises bath correlation functions to incorporate the environmental influence on the system dynamics. Following the procedure for obtaining an exact solution, the correlation in Eq. (9) can be expressed as $C(t)=C_{R}(t)+iC_{I}(t)$, where the real and imaginary components are decomposed as described in Ref. [19].

(18) $$C_{R}(t)=\sum_{\beta =1}^{N_{R}}\;c_{\beta }^{R}\;e^{-\gamma_{\beta }^{R}\;t},\;\;\;\;\;\;\;\;\;\;\text{and}\;\;\;\;\;\;\;\;\;\;C_{I}(t)=\sum_{\beta =1}^{N_{I}}\;c_{\beta }^{I}\;e^{-\gamma_{\beta }^{I}\;t}.$$

This formalism is structured as a hierarchy of coupled equations for density operators, together with auxiliary density operators (ADOs) that enable an accurate representation of memory effects and non-Markovian dynamics, as shown

(19) $$\begin{array}{ll} \frac{d}{dt}{\hat{\rho }}_{n}(t) & \;=(\hat{L}-\sum_{\alpha =R,I}\sum_{\beta =1}^{N_{\alpha }}n_{\alpha \beta }\gamma_{\beta }^{\alpha }){\hat{\rho }}_{n}(t)-i\sum_{\alpha =R,I}\sum_{\beta =1}^{N_{\alpha }}{\hat{L}}^{\times }{\hat{\rho }}_{n_{\alpha \beta }^{+}}(t)\\ & \;-i\sum_{\beta =1}^{N_{R}}c_{\beta }^{R}n_{R\beta }{\hat{L}}^{\times }{\hat{\rho }}_{n_{R\beta }^{-}}(t)+\sum_{\beta =1}^{N_{I}}c_{\beta }^{I}n_{I\beta }{\hat{L}}^{\circ }{\hat{\rho }}_{n_{I\beta }^{-}}(t),\; \end{array}$$

where the notation ${\hat{L}}^{\times }∙=[\hat{L},∙]$ and ${\hat{L}}^{\circ }∙=\{\hat{L},∙\}$, $n=(n_{R1},n_{R2},\ldots ,n_{RN_{R}},n_{I1},n_{I2},\ldots ,n_{IN_{I}})$ is a multi-index of integers $n_{\alpha \beta }\in \{0,N_{c}\}$, $N_{c}$ is the cut-off parameter chosen for convergence, $n_{\alpha \beta }^{\pm }=(n_{\alpha 1},n_{\alpha 2},\ldots ,n_{\alpha \beta -1},n_{\alpha \beta }\pm 1,n_{\alpha \beta +1},\ldots ,n_{\alpha N_{\alpha }})$, and $\hat{L}$ is the Liouvillian operator defined by [19]

(20) $$\hat{L}∙=-\frac{i}{\hbar }{\hat{H}}_{\text{S}}^{\times }∙+{\bar{\Gamma }}_{T}[2\hat{L}∙{\hat{L}}^{†}-{\left({\hat{L}}^{†}\hat{L}\right)}^{\circ }∙].$$

The state labelled n=0=(0,0,…,0) represents the reduced density matrix. Operators with non-zero indices are referred to as ADOs, i.e., n=1={(1,0,…,0),(0,1,…,0),…,(0,0,…,1)}, but are not physical density operators in the conventional sense. In this study, the HEOM is employed as the principal benchmark for comparison with the stochastic Schrödinger equation and the Redfield master equation, owing to its accuracy and versatility in modelling the dynamics of open quantum systems.

### 2.3 The stochastic Langevin-Itô equation

For an open quantum system described by a density operator $\hat{\rho }(t)$, an initial pure state generally evolves into a mixed state due to its interaction with the environment. Consequently, no deterministic equation governs the evolution of the corresponding pure state $\left|\varphi_{\xi }(t)\right\rangle$; instead, its time dependence must be described statistically. Owing to the stochastic fluctuations introduced by the bath, the state follows a stochastic differential equation whose Itô form [32] specifies the infinitesimal variation over a time interval dt:

(21) $$|d\varphi_{\xi }(t)\rangle =|v\rangle \;dt+\sum_{\mu }|u_{\mu }\rangle \;d\xi_{\mu }.$$

Here, |v⟩ is the drift term, while the fluctuations are encoded in the complex Wiener increments $d\xi_{\mu }$, which possess independent real and imaginary components and satisfy [31], [32]

(22) $$M(d\xi_{\mu })=M(d\xi_{\mu }d\xi_{\nu })=0,\;M(d\xi_{\mu }^{*}d\xi_{\nu })=2\delta_{\mu \nu }\;dt,$$

where M(⋅) denotes the ensemble average.

The drift and stochastic terms can be obtained from the master equation for $\hat{\rho }(t)$ under the assumption that the density operator is generated as the stochastic average of pure states:

(23) $$\hat{\rho }(t)=M(|\varphi_{\xi }(t)\rangle \langle \varphi_{\xi }(t)|).$$

The drift term is then given by

(24) $$|v\rangle =(\frac{d\hat{\rho }(t)}{dt})|\varphi (t)\rangle -[\frac{1}{2}\langle \varphi (t)|\frac{d\hat{\rho }(t)}{dt}|\varphi (t)\rangle +ic]|\varphi (t)\rangle ,$$

where ic is an unobservable phase term and $\hat{I}$ denotes the identity operator. The stochastic components arise from the projection of $\frac{d}{dt}\hat{\rho }(t)$ onto the subspace orthogonal to $\left|\varphi_{\xi }(t)\right\rangle$:

(25) $$(\hat{I}-\hat{\rho }(t))(\frac{d\hat{\rho }(t)}{dt})(\hat{I}-\hat{\rho }(t))=2\sum_{\mu }|u_{\mu }\rangle \langle u_{\mu }|.$$

Using (24), (25), the above construction applies to any first-order linear master equation. Substituting the Redfield-type generator from Eq. (7), we obtain the corresponding stochastic Langevin–Itô equation:

(26) $$\begin{array}{ll} \frac{d}{dt}|\varphi_{\xi }(t)\rangle & \;=-\frac{i}{\hbar }{\hat{H}}_{\text{S}}|\varphi_{\xi }(t)\rangle \\ & \;-\sum_{\mu }[{\hat{\zeta }}_{\mu }({\hat{L}}_{\mu };\ell_{\mu }(t))+{\hat{\chi }}_{\mu }({\hat{L}}_{\mu };\ell_{\mu }(t))]|\varphi_{\xi }(t)\rangle .\;\; \end{array}$$

where ${\hat{\zeta }}_{\mu }({\hat{L}}_{\mu };\ell_{\mu }(t))$ is the continuous measurement process as

(27) $${\hat{\zeta }}_{\mu }({\hat{L}}_{\mu };\ell_{\mu }(t))=\gamma_{\mu }(\omega ,t)\;|{\hat{L}}_{\mu }-\ell_{\mu }(t){|}^{2},$$

and ${\hat{\chi }}_{\mu }({\hat{L}}_{\mu };\ell_{\mu }(t))$ is the feedback term as

(28) $${\hat{\chi }}_{\mu }({\hat{L}}_{\mu };\ell_{\mu }(t))=\gamma_{\mu }(\omega ,t)({\hat{L}}_{\mu }^{†}\ell_{\mu }(t)-{\hat{L}}_{\mu }\ell_{\mu }^{*}(t)),$$

and $\ell_{\mu }(t)$ the stochastic measurement variable

(29) $$\ell_{\mu }(t)\;dt=\langle {\hat{L}}_{\mu }\rangle dt+d\xi_{\mu }(t)/\sqrt{\gamma_{\mu }(\omega ,t)}.$$

To analyse the induced dynamics in a geometric form, we define the stochastic density operator and the associated Bloch vector as

(30) $${\hat{\rho }}_{\xi }(t)=|\varphi_{\xi }(t)\rangle \langle \varphi_{\xi }(t)|\;\;\;\;\;\text{and}\;\;\;\;\;{\vec{n}}_{\xi }(t)=\text{Tr}\;\left[\hat{\vec{\sigma }}{\hat{\rho }}_{\xi }(t)\right],$$

or equivalently,

(31) $${\hat{\rho }}_{\xi }(t)=\rho_{\xi }^{\left(0\right)}(t)\hat{I}+\frac{1}{2}\;{\vec{n}}_{\xi }(t)\cdot \vec{\sigma }\;\text{with}\;|{\vec{n}}_{\xi }(t){|}^{2}=1.$$

Substituting Eq. (26) yields the stochastic equations of motion (see Supplementary Note 5):

(32) $$\frac{d}{dt}\rho_{\xi }^{\left(0\right)}(t)={\overset{↔}{\eta }}_{\xi }(t)\cdot {\vec{n}}_{\xi }(t)+C_{\xi }(t)\rho_{\xi }^{\left(0\right)}(t),$$

and

(33) $$\begin{array}{l} \frac{d}{dt}{\vec{n}}_{\xi }(t)={\vec{B}}_{\xi }(t)\times {\vec{n}}_{\xi }(t)+{\overset{↔}{\eta }}_{\xi }(t)\cdot {\vec{n}}_{\xi }(t)+{\vec{T}}_{\xi }(t). \end{array}$$

Averaging over the Wiener processes recovers the deterministic equation of motion in Eq. (14), where the corresponding “corridor path” is defined as

(34) $${\vec{n}}^{\left(av\right)}(t)=M(\;{\vec{n}}_{\xi }(t)\;).$$

The stochastic state $\left|\varphi_{\xi }(t)\right\rangle$ obtained from Eq. (26) may also be represented in the spin-coherent basis,

(35) $$|z\rangle =\frac{1}{\sqrt{1+|z{|}^{2}}}(|+\rangle +z|-\rangle ),$$

where z=x+iy parametrises a stereographic projection of points on the Bloch sphere. Considering a discrete sequence of time steps and inserting the resolution of the identity for coherent states, $\int d^{2}z\;|z\rangle \langle z|=\hat{I}$, yields the formal chain operator representation

(36) $$\psi_{\xi }(z,t)=\langle z\;|\;\varphi_{\xi }(t)\rangle =\langle z\;|\;\hat{K}(t;\xi (t))\;|\varphi_{\xi }(0)\rangle ,$$

with an initial state of the system $\left|\varphi_{\xi }(0)\right\rangle$. The chain operator is given as

(37) $$\hat{K}(t;\xi (t))=\int D[\vec{n}(t)]\;w[\vec{n}(t);\xi (t)]\;e^{\frac{i}{\hbar }S_{0}[\vec{n}(t)]}|z(t)\rangle \langle z(0)|,$$

and $w[\vec{n}(t);\xi (t)]$ is a weighting functional [4], [37],

(38) $$\begin{array}{l} w[\vec{n}(t);\xi (t)]=\prod_{\mu }\exp[-\int_{0}^{t}dt^{\prime }(\zeta_{\mu }[\vec{n}(t^{\prime });\ell_{\mu }(t^{\prime })]+\chi_{\mu }[\vec{n}(t^{\prime });\ell_{\mu }(t^{\prime })])]. \end{array}$$

The relation between the complex parameter z and the position $\vec{n}=(n_{x},n_{y},n_{z})$ on Bloch sphere follows from the stereographic projection:

(39) $$(x,\;y)=\left(\frac{n_{x}}{1-n_{z}},\frac{n_{y}}{1-n_{z}}\right)$$

as used in Refs. [4], [38], [39] (see Supplementary Note 6).

In an alternative geometric formulation, the influence of the environment on a quantum system is represented through a constrained family of admissible trajectories. Within this viewpoint, a general von Neumann–type measurement [40] may be interpreted by comparing the actual path of the measured variable, $\vec{n}(t)$, with a corresponding corridor trajectory that encodes the environment-induced constraint. Quantum coherence between the associated substates—visualised as paths on the Bloch sphere—is suppressed by the weighting functional $w[\vec{n}(t);\ell (t)]$, which declines deviations of an arbitrary path $\vec{n}(t)$ from its corridor counterpart ${\vec{n}}_{\xi }(t)$, as specified by the governing equation,

(40) $$\Delta {\text{n}}_{\eta }(t)=|\;(\vec{n}(t)-{\vec{n}}_{\xi }(t))\cdot {\hat{e}}_{\eta }\;|\gg \;\delta_{\eta }(t)\;\;\;\text{and}\;\;\;w[\vec{n}(t);\xi (t)]\to \;\;0.$$

The corridor width $\delta_{\eta }(t)$ (with η=x,y,z) characterises the environmentally induced uncertainty associated with the components of the basis states |1⟩ and |2⟩, and is defined as [4]

(41) $$\delta_{\eta }(t)={\left[\sum_{\mu }S_{\eta \mu }^{*}\;\gamma_{\mu }(\omega ,t)\;S_{\mu \eta }\right]}^{-1/2},$$

where $S_{\mu \eta }$ denotes the relevant elements of the transformation matrix (see Supplementary Note 1).

We define the *quantum–to–classical crossover time* $\tau_{c}^{\left(\eta \right)}$ as the earliest time at which the stochastic–corridor width falls below a prescribed fraction of its initial quantum spread, i.e.,

(42) $$\tau_{c}^{\left(\eta \right)}=\min\left\{\;t\;|\;\left\|\frac{\partial \delta_{\eta }}{\partial t}\right\|<\varepsilon \;\vee \;\delta_{\eta }(t)=0\right\},$$

where ε is threshold parameter. At this point, transverse quantum fluctuations become strongly suppressed and the system’s evolution becomes confined to a narrow, near–deterministic dynamical corridor.

Eq. (26) does not admit a closed–form exact solution in general, due to its nonlinear and stochastic character on the spin–coherent manifold. Instead, the physically relevant dynamics are captured by a restricted family of paths around the stochastic reference trajectory ${\vec{n}}_{\xi }(t)$, whose statistical weight is governed by the functional $w[\vec{n}(t);\ell (t)]$. Within this framework, the stochastic wavefunction can be approximated as

(43) $$\psi_{\xi }(z,t)=\psi_{0}(z_{\xi }(t),t)\exp\;[-\sum_{\mu }\int_{0}^{t}dt^{\prime }(\zeta_{\mu }[\vec{n};\ell_{\mu }(t^{\prime })]+\chi_{\mu }[\vec{n};\ell_{\mu }(t^{\prime })])].$$

Here the complex parameter $z_{\xi }(t)=x_{\xi }(t)+i\;y_{\xi }(t)$ associated with the stochastic trajectory is related to the Bloch vector ${\vec{n}}_{\xi }(t)=({\bar{n}}_{x}(t),{\bar{n}}_{y}(t),{\bar{n}}_{z}(t))$ via the stereographic projection as Eq. (39). The reference solution $\psi_{0}(z,t)$ corresponds to the environment–free (unitary) evolution generated by the coherent Hamiltonian in Eq. (15), which defines the central of the dynamical corridor,

(44) $$\psi_{0}(z,t)=\psi_{0}(0)\;\exp\;\left[-\frac{i}{2}\int_{0}^{t}dt^{\prime }\;{\vec{B}}_{0}\cdot \vec{n}(t^{\prime })\right],$$

where ${\vec{B}}_{0}$ is given by Eq. (15) in the absence of environmental coupling and $\psi_{0}(0)$ denotes the initial state at t=0.

It must be emphasised that, at this stage, we establish only the equivalence between von Neumann–type measurement amplitudes and restricted sums over paths, rather than a full dynamical derivation. Here $\Delta {\text{n}}_{\eta }(t)$ represents transverse deviations from the stochastic reference path on the spin–coherent manifold. The exponential factor suppresses large deviations, ensuring that only paths confined within the corridor contribute appreciably to the dynamics. Within this framework, an arbitrary path on the Bloch sphere is assumed to take the form

(45) $$\vec{n}(t)={\vec{n}}_{\xi }(t)+\kappa \;\delta \vec{n}(t),\;\delta \vec{n}(t)\equiv \frac{d}{dt}\vec{n}(t),$$

where ${\vec{n}}_{\xi }(t)$ denotes the stochastic (corridor) path and $\delta \vec{n}(t)$ represents a local fluctuation about this path, corresponding to the instantaneous tangent (velocity) of a discretised path segment. The dimensionless parameter κ controls the effective time step Δt associated with the path discretisation. The admissible paths are confined to a finite neighbourhood –the dynamical corridor–surrounding the stochastic reference path, such that

(46) $$\kappa \;|\delta \vec{n}(t)\cdot {\hat{e}}_{\eta }|\ll \delta_{\eta }(t).$$

This condition ensures that arbitrary paths remain within the corridor width $\delta_{\eta }(t)$ and justifies the restricted path–sum representation of quantum state transitions. In the opposite regime, $\kappa \;|\delta \vec{n}(t)\cdot {\hat{e}}_{\eta }|≳\delta_{\eta }(t)$, the weighting functional strongly suppresses such contributions. Paths exhibiting large deviations acquire rapidly decaying (typically exponential) statistical weight and therefore become negligible in the restricted path sum. In this practical sense, large–deviation trajectories effectively “disappear” from the dynamics, and the evolution is dominated by the narrow corridor surrounding ${\vec{n}}_{\xi }(t)$.

The progressive suppression of large–deviation trajectories provides a natural criterion for defining the quantum–to–classical crossover time. As the system evolves, environmental monitoring continuously narrows the dynamical corridor by reducing the corridor width $\delta_{\eta }(t)$, eventually confining the dynamics to the stochastic reference path ${\vec{n}}_{\xi }(t)$.

## 3 Results of numerical simulation and modelling

### 3.1 Comparative methodologies

This work examines quantum state transitions in the spin–boson model from a continuous–measurement perspective, focusing on how the choice of system–bath coupling operators shapes the interplay between external driving and environmental backaction. Our central goal is to determine when coherent dynamics and population transfer can be sustained by the external field, and when measurement–induced decoherence associated with the coupling operators dominates and suppresses coherence.

To this end, we consider two representative classes of system bath coupling operators that capture distinct physical mechanisms. The first,

(47) $${\hat{L}}_{T}=c\;{\hat{\sigma }}_{x},$$

corresponds to a transverse, transition–inducing (dipole–like) coupling, which promotes population transfer between the two levels and is commonly associated with relaxation processes. The second,

(48) $${\hat{L}}_{AL}=\sum_{m=1}^{2}c_{m}\;{\hat{\sigma }}_{m}^{+}{\hat{\sigma }}_{m}^{-},$$

represents a local site–resolved (diagonal) coupling that induces site dependent dephasing. Asymmetric environmental effects are introduced through unequal coupling strengths satisfying $g_{k}^{\left(2\right)}=\text{r}g_{k}^{\left(1\right)}$, which mimic pigment specific exciton–phonon interactions observed in photosynthetic energy–transfer complexes.

The environment is modelled using an under-damped Brownian spectral density, as defined in Eq. (10), which provides a minimal yet flexible description of structured vibrational environments. This bath model allows us to explore the combined influence of coupling strength, vibrational resonance, and damping on the open system dynamics. The quantum dynamics are computed and cross validated using three complementary approaches:

1. **Redfield master equation** in Lindblad form, from which a Bloch–vector equation of motion is derived. This formulation enables a geometric decomposition of the dynamics into coherent precession, dissipative relaxation, and bath–induced torque contributions.
2. **Hierarchical Equations of Motion (HEOM)**, employed as a numerically exact benchmark spanning the weak– to strong–coupling regimes. HEOM calculations are performed using the open–source QuTiP library [19], which provides a reliable platform for modelling bosonic environments within the spin–boson framework.
3. **Stochastic Langevin–Itô (stochastic Schrödinger) equation**, which generates ensembles of quantum trajectories. The statistical properties of these trajectories are encoded in a weighting functional, giving rise to a finite stochastic “corridor width” that constrains the admissible paths on the (generally non–unitary) Bloch sphere.

### 3.2 Steady time and steady probability

**Fig. 2:**
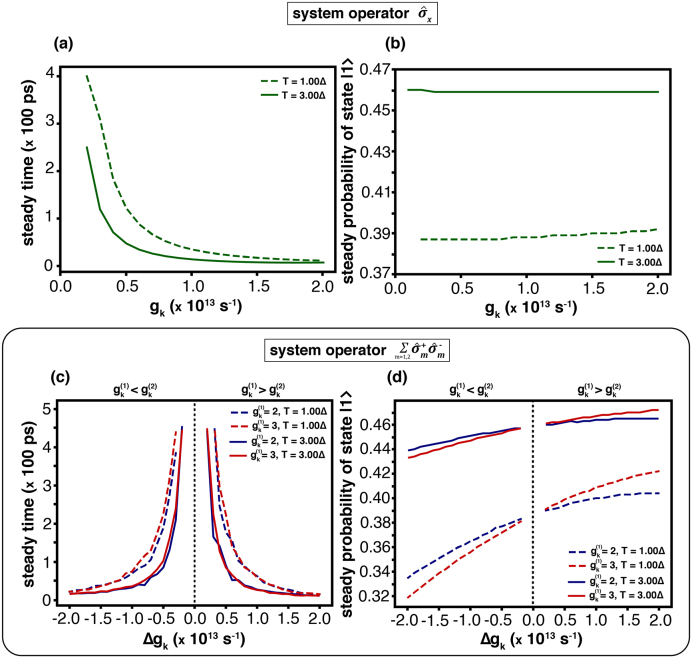
Steady time $\left(\tau_{\text{sd}}\right)$ and steady probability $\left(P_{\text{sd}}\right)$ are shown for temperatures T=1.00Δ (solid lines) and T=3.00Δ (dashed lines). For the system operator ${\hat{L}}_{T}$: (a) steady time as a function of $g_{k}$, and (b) steady probability of state |1⟩. For the system operator ${\hat{L}}_{AL}$: (c) steady time versus $\Delta g_{k}=g_{k}^{\left(1\right)}-g_{k}^{\left(2\right)}$—blue line for $g_{k}^{\left(1\right)}=2.0\Delta ,\;g_{k}^{\left(2\right)}=[0.0,4.0]\Delta$; red line for $g_{k}^{\left(1\right)}=3.0\Delta ,\;g_{k}^{\left(2\right)}=[1.0,5.0]\Delta$—and (d) corresponding steady probability of state |1⟩.

In this work, the total and systematic Hamiltonians are defined by (1), (3), respectively. The system coupled to a phonon bath is described by the under-damped Brownian spectral density Eq. (10). Numerical simulations are performed through the HEOM formalism following Ref. [19]. To ensure comparison consistency, the system operator in Eq. (4) is fixed, while the coupling parameter $c\;\;(\text{or}\;\;c_{m})$ is varied. This results in effectively tuning the coupling strengths $g_{k}\;\;(\text{or}\;\;g_{k}^{\left(m\right)})$. Parameters related to bath correlation are kept constant throughout, whereas the setting cut off parameter is $N_{c}=5$. From the results by HEOM, the population of state |1⟩ is computed, in order to determine *the steady time* $\left(\tau_{sd}\right)$—the moment at which the probability stabilizes—. The corresponding *steady probability* $\left(P_{sd}\right)$, is shown in Fig. 2. The primary objective is to clarify how the system operator, within the interaction Hamiltonian, governs state-transition dynamics under different coupling configurations (For the definition of $\tau_{sd}$ and $P_{sd}$, please see Supplementary Note 7–7.2).

Fig. 2(a) shows that the steady time is highly sensitive to the coupling strength $g_{k}$ for the system operator ${\hat{\sigma }}_{x}$. It attains large values when $g_{k}$ is small—approximately 4.00(×100ps) at T=1.00Δ and 2.49(×100ps) at T=3.00Δ—while it decreases progressively as $g_{k}$ increases. Such a behaviour indicates that weaker coupling slows the state transition, while stronger coupling enhances it. In the limiting case of $g_{k}=0$, the steady time diverges, implying a complete suppression of transitions. At fixed $g_{k}$, the higher temperatures (T=3.00Δ) consistently yield steady times shorter than lower ones (T=1.00Δ). For this reason, this demonstrates that the thermal excitation accelerates the transition dynamics.

For the system operator ${\hat{L}}_{AL}$, as shown in Fig. 2(c), the dynamics are examined as a function of $\Delta g_{k}=g_{k}^{\left(1\right)}-g_{k}^{\left(2\right)}$. The steady time for $-\Delta g_{k}$ and $+\Delta g_{k}$ is almost symmetric, suggesting that the magnitude $\left|\Delta g_{k}\right|$ governs the behaviour in a manner analogous to $g_{k}$ in the ${\hat{\sigma }}_{x}$ case. For small $\left|\Delta g_{k}\right|$, the system exhibits long steady times—approximately >4.5(×100,ps) at T=1.00Δ and 4.0–4.5(×100,ps) at T=3.00Δ—which gradually shorten as $\left|\Delta g_{k}\right|$ increases. Under the identical coupling and the same temperature, the system operator ${\hat{L}}_{AL}$ consistently yields longer steady times than ${\hat{\sigma }}_{x}$. Therefore, the result indicates a delay in the onset of quantum-state transitions, relative to the ${\hat{\sigma }}_{x}$ interaction.

As shown in Fig. 2(b), the steady probability associated with the system operator ${\hat{\sigma }}_{x}$ remains approximately constant across different values of $g_{k}$. Nonetheless, at the higher temperature (T=3.00Δ), the steady probability—approximately 0.46—is notably greater than the probability at lower temperatures (T=1.00Δ), where it is around 0.39. A comparable tendency is observed for the operator ${\hat{L}}_{AL}$ in Fig. 2(d): at T=3.00Δ, with the probability lies in 0.43–0.47, whereas it decreases to 0.32–0.42 at T=1.00Δ. Moreover, the steady probability is systematically lower in the region $-\Delta g_{k}$ than in $+\Delta g_{k}$. This behaviour arises since $-\Delta g_{k}$ corresponds to $g_{k}^{\left(2\right)}>g_{k}^{\left(1\right)}$, favouring transitions towards the state |2⟩, hence reducing the population of state |1⟩. Conversely, when $\Delta g_{k}>0$ (i.e., $g_{k}^{\left(2\right)}<g_{k}^{\left(1\right)}$), the dynamics favour |1⟩, leading to a higher steady probability.

### 3.3 Analysing the dynamics of state transition through the corridor path

**Fig. 3:**
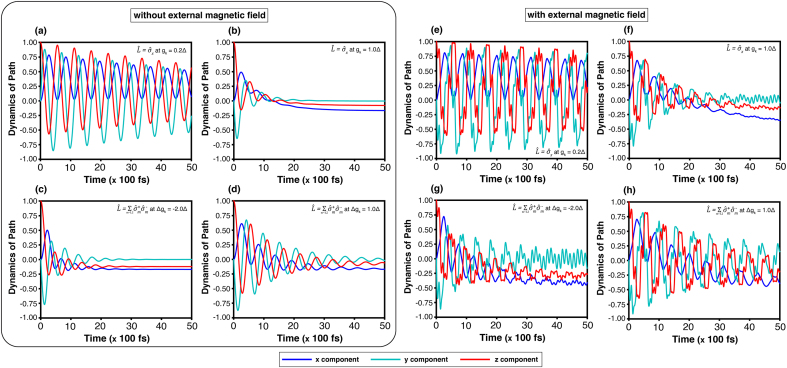
Time evolution of the corridor path on the Bloch sphere, $(n_{x}(t),n_{y}(t),n_{z}(t))$, at temperature T=3.00Δ, obtained using the HEOM. Results are shown for cases without an external magnetic field: system operator ${\hat{\sigma }}_{x}$ with (a)$g_{k}=0.2\Delta$ and (b)$g_{k}=1.0\Delta$; and system operator ${\hat{L}}_{AL}$ with (c)$\Delta g_{k}=-2.0\Delta$ and (d)$\Delta g_{k}=1.0\Delta$. Corresponding results with an external magnetic field ($B_{0}=2.0\Delta$, $\omega_{B}=5.0\Delta$) are shown for ${\hat{\sigma }}_{x}$ with (e)$g_{k}=0.2\Delta$ and (f)$g_{k}=1.0\Delta$, and for ${\hat{L}}_{AL}$ with (g)$\Delta g_{k}=-2.0\Delta$ and (h)$\Delta g_{k}=1.0\Delta$.

Fig. 3 presents the time evolution of the corridor path components $n_{x}(t)$, $n_{y}(t)$, and $n_{z}(t)$ which are obtained through the HEOM method. For the same system operator, the coupling constant $g_{k}$ (or $\Delta g_{k}$) and temperature (T=3.00Δ) are kept fixed, while the comparison highlights the effects of the external magnetic field.

Figs. 3(a)–(d) exemplify the trajectories on the Bloch sphere without an external magnetic field. The result from the system operator ${\hat{\sigma }}_{x}$ and coupling $g_{k}=1.0\Delta$ as shown in Fig. 3(b), reaches a steady state approximately at 30(×100,fs), which is faster than the case of weaker coupling $g_{k}=0.2\Delta$ as shown in Fig. 3(a). Similarly, the result from the system operator ${\hat{L}}_{AL}$ with $|\Delta g_{k}|=2.0\Delta$ as shown in Fig. 3(c), reaches a steady state around 40(×100,fs), which is also faster than the case of $|\Delta g_{k}|=1.0\Delta$ as shown in Fig. 3(d). Despite different coupling strengths, the steady-state values of both system operators—between ${\hat{\sigma }}_{x}$ with $g_{k}=1.0\Delta$ and ${\hat{L}}_{AL}$ with $|\Delta g_{k}|=1.0\Delta$—are nearly identical. This agrees with the steady-state probabilities at high temperature (T=3.00Δ), where Fig. 2(b) gives $P_{\text{sd}}=0.46$ for $g_{k}=1.0\Delta$ and Fig. 2(d) shows $P_{\text{sd}}\approx 0.46$ for $\Delta g_{k}=1.0\Delta$.

Figs. 3(e)–(h) show the effect of the external magnetic field, which introduces an additional oscillation whose frequency matches the intrinsic phase frequency. Such an effect leads to periodic fluctuations synchronized with the driving field. Comparing the dynamic components $n_{x}(t)$ and $n_{z}(t)$ between the driven and undriven cases, it reveals that both quantities exhibit reduced amplitudes under the influence of the external field.

#### 3.3.1 The geometric decomposition of equation of motion without an external magnetic field

**Fig. 4:**
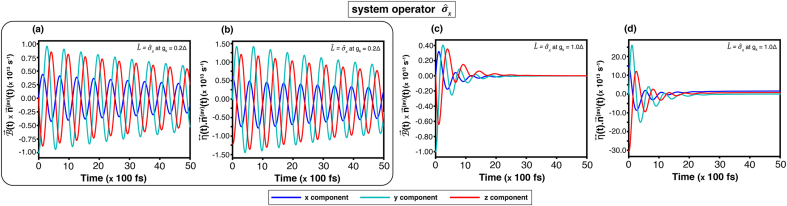
Time evolution of the equation of motion governed by Eq. (14) for the system operator ${\hat{\sigma }}_{x}$ at temperature T=3.00Δ. Results are shown for coupling strengths $g_{k}=0.2\Delta$ ((a)-(b)) and $g_{k}=1.0\Delta$ ((c)-(d)) without an external magnetic field. Panels (a)–(c) show the precession term $\vec{B}(t)\times {\vec{n}}^{\left(av\right)}(t)$, and (b)–(d) the relaxation term $\overset{↔}{\eta }(t)\cdot {\vec{n}}^{\left(av\right)}(t)$.

The state-transition dynamics of the corridor path, which is obtained from the equation of motion of the quantum trajectory on the Bloch sphere Eq. (14) are examined. For the weak coupling ($g_{k}=0.2\Delta$), Fig. 2(a) shows a long steady time approximately at 2.5(×100ps). It indicates that the transition exhibits strong fluctuations and requires relatively much time to reach a dynamical equilibrium. Such a result agrees with the precession and relaxation components in Fig. 4(a) and 4(b), which oscillate during 0–50(×100fs). On the contrary, when the coupling increases to $g_{k}=1.0\Delta$, the steady time in Fig. 2(c) decreases noticeably, which implies that the transition reaches an equilibrium faster. Thankfully, it is consistent with Fig. 4(b) and (d), showing the corresponding precession and relaxation terms, which stabilize within ∼30(×100fs).

**Fig. 5:**
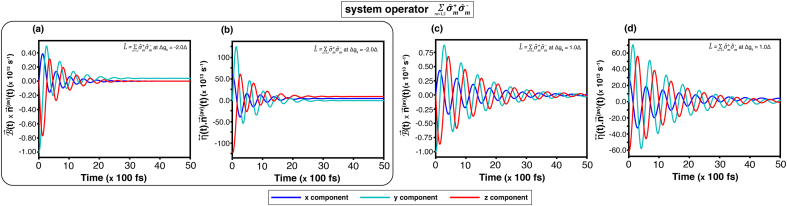
Time evolution of the equation of motion governed by Eq. (14) for the system operator ${\hat{L}}_{AL}$ at temperature T=3.00Δ. Results are shown for coupling strengths $\Delta g_{k}=-2.0\Delta$ ((a)–(b)) and $\Delta g_{k}=1.0\Delta$ ((c)–(d)) without an external magnetic field. Panels (a)–(c) show the precession term $\vec{B}(t)\times {\vec{n}}^{\left(av\right)}(t)$, and (b)–(d) the relaxation term $\overset{↔}{\eta }(t)\cdot {\vec{n}}^{\left(av\right)}(t)$.

For the system operator ${\hat{L}}_{AL}$, the result conforms to the previous analysis. As shown in Fig. 2(c) at high temperature (T=3.00Δ), the steady time at $|\Delta g_{k}|=2.0\Delta$ is shorter than that at $|\Delta g_{k}|=1.0\Delta$. Confirming a faster convergence. This trend is corroborated by the precession and relaxation components in Fig. 5(a) and (b) compared with Fig. 5(c) and (d), which display correspondingly quicker damping and stabilization.

#### 3.3.2 The equation of motion with an external magnetic field

The influence of an external magnetic field was analysed through the equation of motion on the Bloch sphere Eq. (14).

**Fig. 6:**
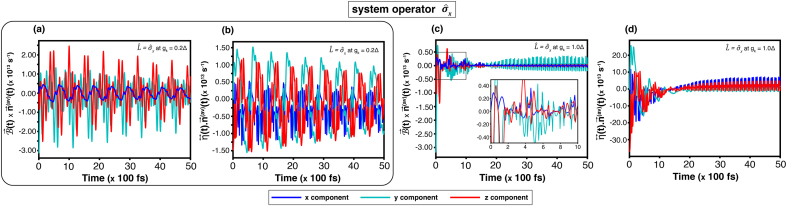
Time evolution of the equation of motion governed by the equation of motion Eq. (14). For the system operator ${\hat{\sigma }}_{x}$ at temperature T=3.00Δ, results are shown for coupling strengths $g_{k}=0.2\Delta$ ((a)-(b)) and $g_{k}=1.0\Delta$ ((c)-(d)) under an external magnetic field of $B_{0}=2.0\Delta$ and $\omega_{B}=5.0\Delta$. Panels (a)–(c) show the precession term $\vec{B}(t)\times {\vec{n}}^{\left(av\right)}(t)$, and (b)–(d) the relaxation term $\overset{↔}{\eta }(t)\cdot {\vec{n}}^{\left(av\right)}(t)$.

For the system operator ${\hat{\sigma }}_{x}$, the precession components which are shown in Fig. 6(a) and (c) for $g_{k}=0.2\Delta$ and $g_{k}=1.0\Delta$, respectively, reveal that the y- and z-components exhibit stronger fluctuations than the x-component. These oscillations arise from the driving frequency of the external magnetic field. However, the overall result resembles the figures from the field-free case in Fig. 4(a) and (b).

**Fig. 7:**
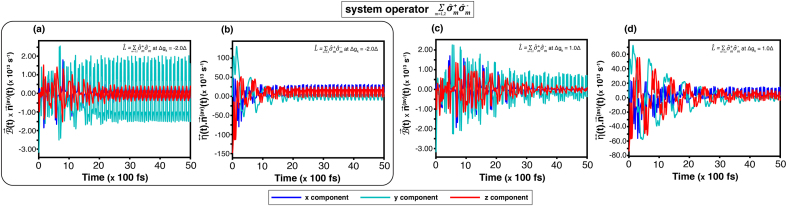
Time evolution of the equation of motion governed by the equation of motion Eq. (14). For the system operator ${\hat{L}}_{AL}$ at temperature T=3.00Δ, results are shown for coupling strengths $\Delta g_{k}=-2.0\Delta$ ((a)-(b)) and $\Delta g_{k}=1.0\Delta$ ((c)-(d)) under an external magnetic field of $B_{0}=2.0\Delta$ and $\omega_{B}=5.0\Delta$. Panels (a)–(c) show the precession term $\vec{B}(t)\times {\vec{n}}^{\left(av\right)}(t)$, and (b)–(d) the relaxation term $\overset{↔}{\eta }(t)\cdot {\vec{n}}^{\left(av\right)}(t)$.

For the system operator ${\hat{L}}_{AL}$, Fig. 7(a) and (b) display a comparable response: the external-field frequency induces additional oscillations in the precession dynamics relative to Fig. 5(a) and (b). In all the four cases, the y- and z-components attain larger amplitudes than the components in the field free condition. Nevertheless, the x-component remains nearly unchanged.

The relaxation terms, as Figs. 6(b, d) and 7(b, d), maintain magnitudes similar to those without the external field, though oscillatory features arise from the magnetic-field frequency. In the case of ${\hat{L}}_{AL}$, the fluctuation also depends on $\left|\Delta g_{k}\right|$. Therefore, a phase reversal is consistent with the discussion in Section 3.3.1.

### 3.4 Analysing the dynamics of state transition through the stochastic path

#### 3.4.1 Weighting functional

**Fig. 8:**
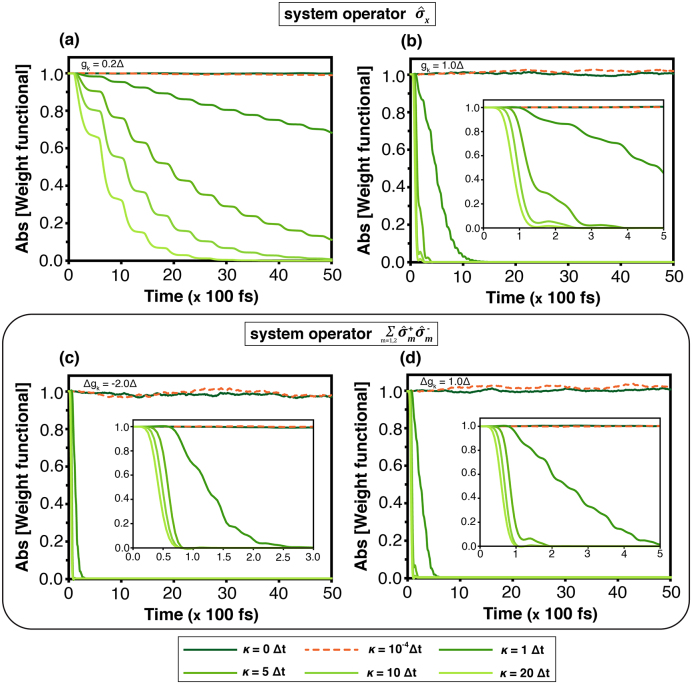
The weighting functional derived from Eq. (38) is shown for an arbitrary path $\vec{n}(t)$ given in Eq. (45) in the absence of an external magnetic field at temperature T=3.00Δ. The parameter $\kappa \in \{0,\;10^{-4},\;1,\;5,\;10,\;20\}\Delta t$ is varied for two forms of the system operator: ${\hat{L}}_{T}$ in (a)$g_{k}=0.2\Delta$ and (b)$g_{k}=1.0\Delta$, and ${\hat{L}}_{AL}$ in (c)$\Delta g_{k}=-2.0\Delta$ and (d)$\Delta g_{k}=1.0\Delta$.

The weighting functional, derived from Eq. (38), is a mathematical quantity associated with probability distributions and density operators. It quantifies the dynamical deviation between an arbitrary path and the corridor path, thus reflecting the interaction between them. In this study, the corridor path, ${\vec{\text{n}}}^{\left(av\right)}(t)$, is defined from the solution of the stochastic Schrödinger equation Eq. (34), which is consistent with ${\vec{\text{n}}}^{\left(av\right)}(t)$ obtained from the HEOM approach. This calculation is verified by the population of state |1⟩ at high temperature, T=3.00Δ (see Supplementary Note 7–7.1). The arbitrary path in Eq. (45) is generated by varying κ. Parameter κ=0 corresponds to the corridor path, i.e., $\vec{\text{n}}(t)={\vec{\text{n}}}^{\left(av\right)}(t)$. Fig. 8 presents the absolute value of the weighting functional for κ∈{0,1,5,10,20}Δt (The less intense green, the higher κ)

In Figs. 8(a) and (b), the results correspond to the system operator ${\hat{\sigma }}_{x}$ at $g_{k}=0.2\Delta$ and $g_{k}=1.0\Delta$, whereas Figs. 8(c) and (d) show the system operator ${\hat{L}}_{AL}$ at $\Delta g_{k}=-2.0\Delta$ and $\Delta g_{k}=1.0\Delta$, respectively.

The larger values of κ yield more deviation between the arbitrary and corridor paths. For this reason, these figures show a smaller absolute value of the weighting functional where longer κ, that is the less intense green, the smaller absolute value of the weighting functional. The display is consistent with the sense that the more deviation ought to have a lower probability and a smaller weighting functional. However, for the same system operator, the weighting functional (in the deviated paths) at smaller $g_{k}$ (or $\left|\Delta g_{k}\right|$) remains larger than that for higher coupling strengths. This signifies that the higher coupling strengths impose more constraints on the transition dynamics. That is the stronger coupling leads the transition more rapid, from the weighting functional perspective. Based on this auxiliary mathematical quantity, it contributes the novel perspective: the width of the corridor on the Bloch’s sphere.

#### 3.4.2 Width of corridor

**Fig. 9:**
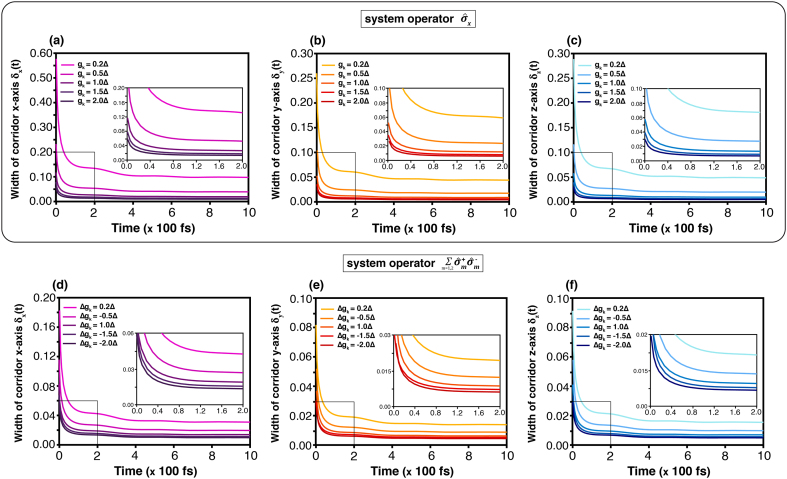
Time evolution of the corridor width defined in Eq. (41) at temperature T=3.00Δ for different values of $g_{k}$ or $\Delta g_{k}$. (a), (b) and (c) show $\delta_{x}(t)$, $\delta_{y}(t)$ and $\delta_{z}(t)$, respectively, for the system operator ${\hat{\sigma }}_{x}$, while (d), (e) and (f) show $\delta_{x}(t)$, $\delta_{y}(t)$ and $\delta_{z}(t)$ for the operator ${\hat{L}}_{AL}$.

The width of the corridor, defined in Eq. (41), depends on the bath through the dissipation coefficient $\Gamma_{\mu }(\omega ,t)$. As shown in Fig. 9(a)–(f), they show a similar tendency for each coupling strength, despite the different types. The simulation shows that the width of every component is high during the early stage, and then decays as time goes on. The wide width signifies the less constraint for the dynamics and superposition, expressing the quantum coherent property. From the result, the state-transition has a quantum coherence property during the early time, but loses this property later. We also find that the high coupling strength makes the width narrower. Hence, the strong coupling increases the quantum decoherence in our model.

**Fig. 10:**
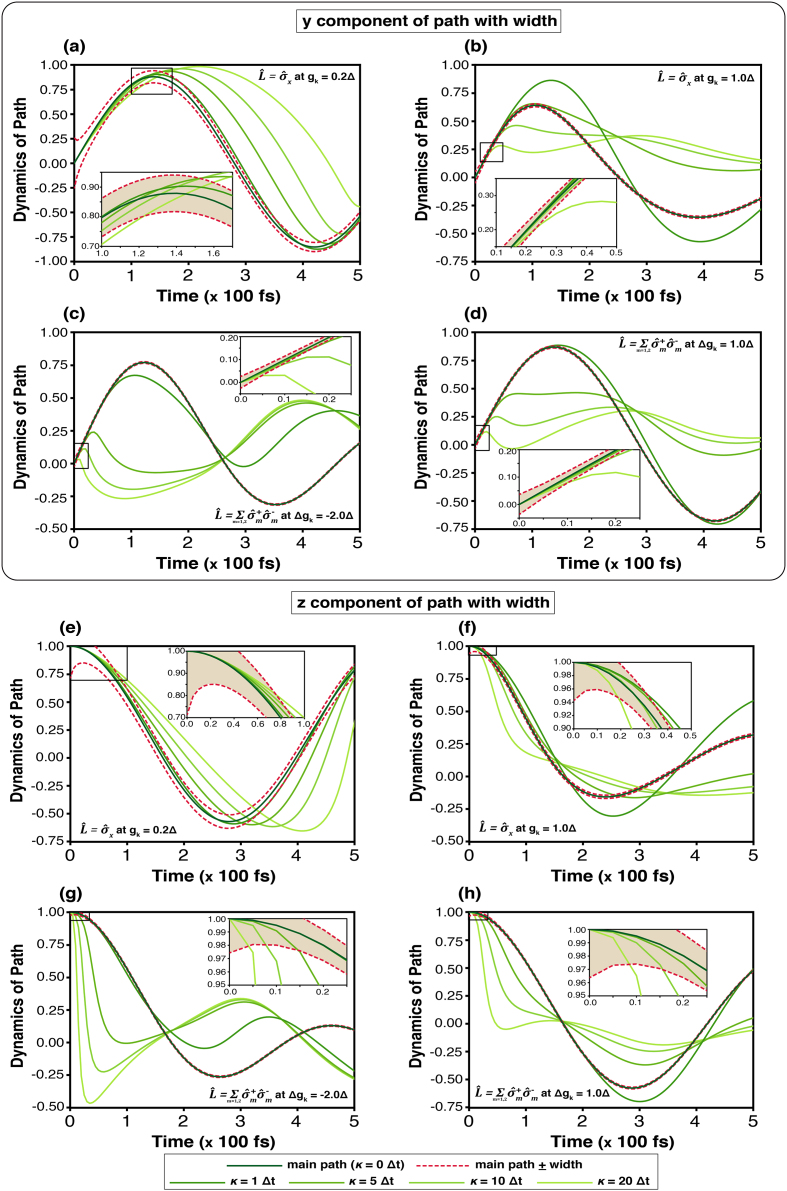
Quantum paths of state transitions on Bloch sphere influenced by the phonon bath in the absence of an external magnetic field. The dark green line denotes the central corridor path (κ=0Δt), while the deep red dashed lines indicate the corridor width. Arbitrary paths are generated following Eq. (45) by varying κ=1Δt (deep green), 5Δt (bright green), 10Δt (light yellow-green), and 20Δt (pale yellow-green). In (a)–(b) and (e)–(f) show $n_{y}(t)$ and $n_{z}(t)$, respectively, for the system operator ${\hat{\sigma }}_{x}$; (c)–(d) and (g)–(h) correspond to ${\hat{L}}_{AL}$.

**Fig. 11:**
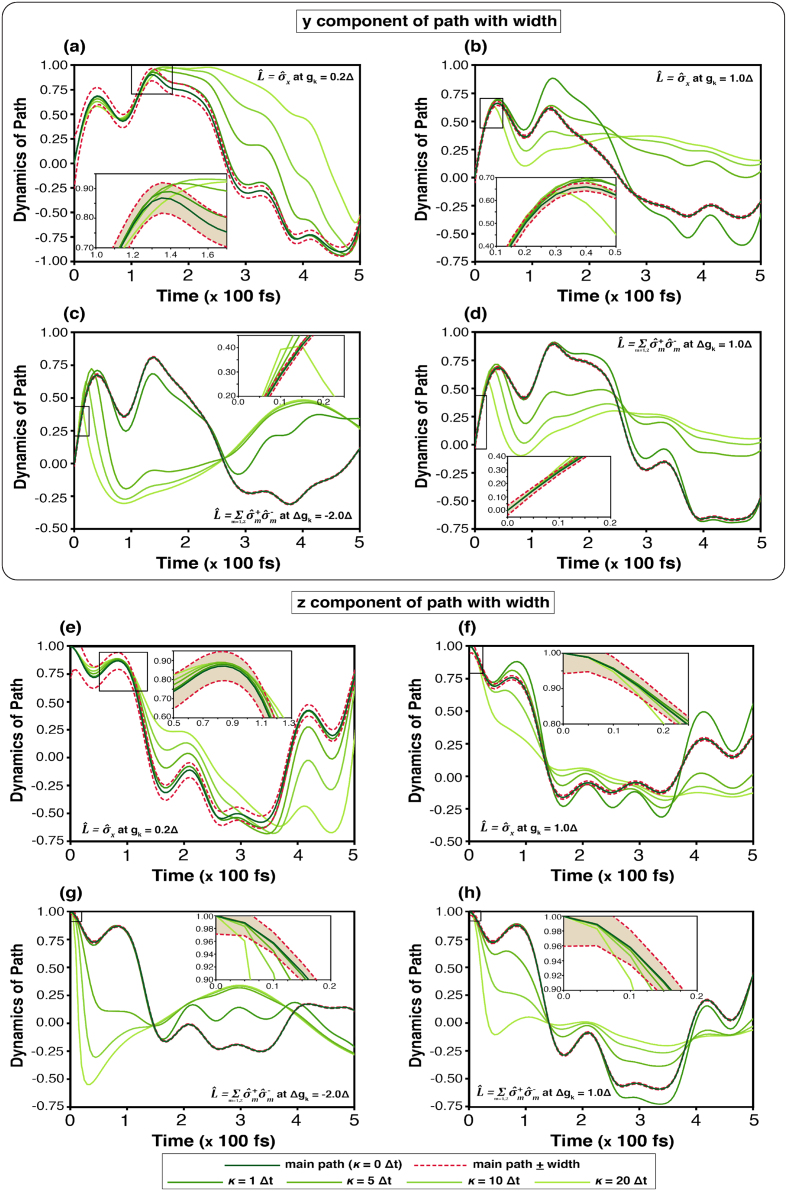
Quantum paths of state transitions on Bloch sphere influenced by the phonon bath under effect of an external magnetic field. The dark green line denotes the central corridor path (κ=0Δt), while the deep red dashed lines indicate the corridor width. Arbitrary paths are generated following Eq. (45) by varying κ=1Δt (deep green), 5Δt (bright green), 10Δt (light yellow-green), and 20Δt (pale yellow-green). In (a)–(b) and (e)–(f) show $n_{y}(t)$ and $n_{z}(t)$, respectively, for the system operator ${\hat{\sigma }}_{x}$; (c)–(d) and (g)–(h) correspond to ${\hat{L}}_{AL}$.

The corridor path, its width, and an arbitrary trajectory are shown in Fig. 10 (No external magnetic field) and Fig. 11 (Applying External magnetic field). These displays resemble a continuous measurement, which constrains the dynamics of the state transition. The simulation displays $n_{y}(t)$ and $n_{z}(t)$, representing respectively the imaginary part of the coherence term of the density matrix and the population difference between the two states |1⟩ and |2⟩. Although the simulation seemingly shows that the trajectory can be outside the width, it is inside the width compulsorily. Thus, the state-transition must be constrained within the width after approaching the width.

According to Eq. (14), the precession term dominates the motion, particularly in the y- and z-components. At the initial stage, the arbitrary path partly lies outside the corridor, with the trajectory at κ=20Δt exhibiting the largest deviation. In the field-free case (Fig. 10), the arbitrary path gradually converges to the corridor path for long times (>30×100fs). Conversely, the difference persists when the system operator is ${\hat{\sigma }}_{x}$ and $g_{k}=0.2\Delta$, because of a longer relaxation time. Under the external field (Fig. 11), the $n_{y}(t)$ components in Fig. 11(a)–(d) show that trajectories (at κ=10Δt and 20Δt) remain largely outside the width—particularly for κ=20Δt—and do not converge to the corridor path but oscillate around zero. Similarly, $n_{z}(t)$ in Fig. 11(e)–(h) initially deviates from the width, but later becomes confined within the width more than $n_{y}(t)$, especially at κ=20Δt. Overall, these figures, which represent a virtual quantum continuous measurement, articulate the constraint and quantum decoherence property for the state-transition.

### 3.5 Quantum-to-classical crossover phenomena in biological systems

This section describes the biological system under consideration, namely the phycobiliprotein PC645, characterised by the system operator ${\hat{L}}_{AL}$. We have simulated the transfer’s time and the trajectory with the width of the corridor for the system. The differences, between the spin-boson and the biological system, involve the temperature, coupling strength, spectral density etc. On this ground, the result is not absolutely the same, yet the overall tendency rather resembles. The variables of the PC645 for the simulation are followed by [41] (see Supplementary Note 7–7.1).

**Fig. 12:**
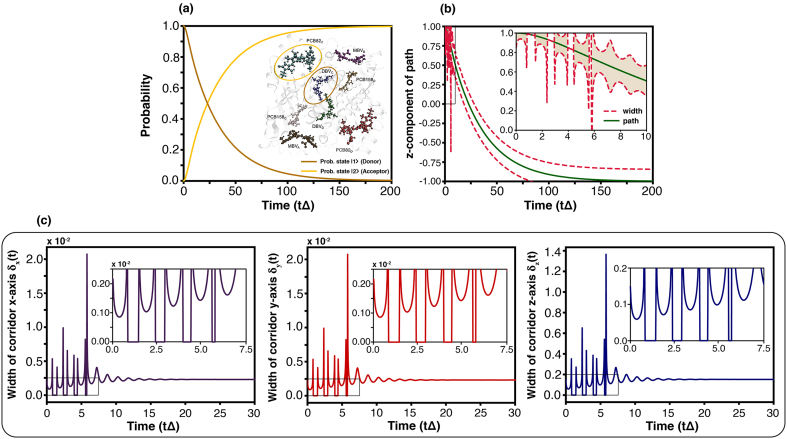
(a) Probability of exciton transfer from dihydrobiliverdins: ${\text{DBV}}_{\text{c}}$ (donor; |1⟩) to phycocyanobilin 82: ${\text{PCB82}}_{\text{c}}$ (acceptor; |2⟩) in the phycobiliprotein PC645 complex [41], visualized by the Visual Molecular Dynamics (VMD) software, version 1.9.3 [42]. The dynamics are modelled using a simplified spin–boson framework with an under-damped Brownian motion spectral density, while adopting PC645 parameters from Ref. [41]. The coupling strengths of the donor and acceptor are set to 180 ${\text{cm}}^{-1}$ and 500 ${\text{cm}}^{-1}$, respectively, with a tunnelling amplitude Δ = 24.3 ${\text{cm}}^{-1}$ and temperature T=300K. (b) Trajectory dynamics on the Bloch sphere for the z-component. The dark green solid line denotes the central corridor path, while the deep red dashed lines indicate the corridor width. (c) Time evolution of the corridor width, as defined in Eq. (41), for the x-, y- and z-components for PC645 complex case.

The Fig. 12(a) provides the transfer’s time with probability. The display shows that the state transfer is almost total. Such a characteristic is plausible due to the strong coupling strength. Therefore, the state-transfer occurs smoothly in nature, making the photosynthesis highly efficient. Moreover, the transfer’s time in our simulation is rather consistent with the work [41]. They observe that the time is in the picosecond range.

Fig. 12(b) portrays the trajectory with the width of the corridor. The specific attribute is the intense width’s oscillation during early time. The simulation portrays the very wide, almost zero and mild width. The very wide signifies the freedom and superposition for the path, summarizing the coherence property. Almost zero insinuates the intense constraint that completely controls the path, called the decoherence property. Lastly the mild width shows semi property. The display shows that these three attributes switch to each other during early stage. For this reason, the biological system has a recoherence property, in which the switching constraint affects the state-transfer coherence back and forth. Nevertheless, this particular character ceases after a specific time. As shown, the width of the corridor has a mild range after the early stage. We discover that this character is found in the biological system, such as the work [4]. The possible factor for this behaviour is the coupling strength and spectral density. In the biological system, its values make the dissipation coefficient oscillate in the early stage.

**Fig. 13:**
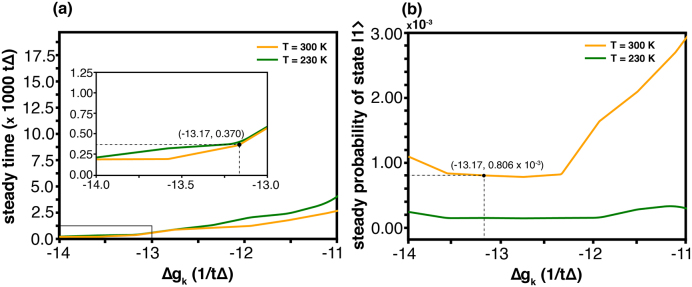
(a) Steady time $\left(\tau_{\text{sd}}\right)$ and (b) steady probability $\left(P_{\text{sd}}\right)$ of exciton transfer from ${\text{DBV}}_{\text{c}}$ (donor; |1⟩) to ${\text{PCB82}}_{\text{c}}$ (acceptor; |2⟩) are shown for temperatures T=230 K (green lines) and T=300 K (orange lines), and vary the coupling strength of acceptor. By −13.17Δ=(180/24.3)Δ−(500/24.3)Δ. (For interpretation of the references to colour in this figure legend, the reader is referred to the web version of this article.)

The Fig. 12(c) shows the width of the corridor for every component. These figures seemingly exhibit the same tendency. However, the scale in the vertical bar is extremely different ($10^{-2}$ for x, y and 1 for z). The result signifies that the constraint has a significant impact on x- and y-components but lessens its effect on z component. Therefore, real nature constrains the coherence property (between initial state and final state) significantly but has less influence on state-transfer. Fig. 13 provides a steady time in the biological system. Its tendency and value are rather similar to Fig. 2(c) which is the case for different coupling values and $g_{k}^{\left(2\right)}>g_{k}^{\left(1\right)}$. This signifies that the biological system exhibits the different coupling strengths for each pigment. Such a characteristic is also found in the spin-boson model in our simulation.

## 4 Discussion

In this work, we examine how different forms of system–bath coupling and an external magnetic field influence the dynamics of the spin–boson model by employing three complementary approaches: the HEOM method, the Redfield master equation, and the stochastic Schrödinger equation. The HEOM technique provides numerically accurate results across weak, intermediate, and strong coupling regimes, although it offers limited physical transparency regarding environmental effects. In contrast, the Redfield master equation—an analytical framework—enables direct evaluation of such effects from the equation of motion, Eq. (14), which describes quantum-state transitions through the Bloch sphere. The stochastic Schrödinger equation, on the other hand, introduces a novel interpretation wherein environmental influences appear as continuous measurements represented by the path integral and weighting functional. All simulations are conducted at high temperature (T=3.00Δ), where the three methods yield comparable probabilities of finding the state |1⟩ (see Supplementary Note 7–7.1). The results clearly demonstrate how the interplay between the system–bath coupling and external magnetic field dictates the evolution of quantum states in the spin–boson model. Across all coupling regimes, both factors significantly modify the amplitude and frequency of oscillations as well as the geometric trajectories of the Bloch vector on the Bloch sphere.

We next analyse the effect of system–bath coupling for two representative interaction types: non-local coupling (system operator ${\hat{L}}_{T}$) and local coupling (system operator ${\hat{L}}_{AL}$). In both cases, the coupling strength ($g_{k}$ or $\left|\Delta g_{k}\right|$) directly influences the quantum-state transition dynamics, as shown in Fig. 2. The characteristic steady time reflects the lifetime of state populations and coherence in the two-level system, consistent with previous studies on coherence preservation and decay in various systems—such as weak and strong bath couplings in photosynthetic complexes [1], [3], [4], [41], cavity tuning for atomic coherence [7], and functionalisation-induced control of molecular coherence [43]. The steady time ($\tau_{sd}$) scales approximately as $\tau_{sd}\propto \lambda^{-2}(T_{0}(\omega )/T)$ at high temperature, where $T_{0}(\omega )=\hbar \omega /k_{B}$ is the Einstein temperature, indicating its dependence on both coupling strength and temperature. For ${\hat{\sigma }}_{x}$ coupling (Fig. 2a), weak coupling ($0\leq g_{k}<1$) yields slower transitions, absent at $g_{k}=0$ and accelerated for $g_{k}>0$, whereas strong coupling ($1\leq g_{k}<2$) induces faster transitions with only minor variation in the steady time. The local-coupling case ${\hat{L}}_{AL}$ (Fig. 2c) exhibits a similar trend, with little sensitivity to variations in $g_{k}^{\left(1\right)}$. At elevated temperature (T=3.00Δ), the steady time for both coupling schemes becomes noticeably longer than at low temperature. The steady probability ($P_{sd}$) for the ${\hat{\sigma }}_{x}$ operator (Fig. 2b) depends primarily on temperature, with negligible influence from $g_{k}$. In contrast, for ${\hat{L}}_{AL}$ coupling (Fig. 2(d)), the steady probability depends on both T and $g_{k}^{\left(m\right)}$: higher $g_{k}^{\left(m\right)}$ values at a given state |m⟩ increase the probability of occupying that state, even when $\Delta g_{k}$ remains constant. Differences in $g_{k}^{\left(m\right)}$ between equivalent states thus lead to distinct dynamical behaviours.

The quantum-state transition dynamics are visualised on the Bloch sphere using the equation of motion (Eq. 14) derived from the Redfield master equation (see Supplementary Note 4). The Bloch sphere is defined with the north and south poles corresponding to the states |1⟩ and |2⟩, respectively. Transitions between these states are governed by variations in $n_{z}(t)$, which reflect the population difference between |1⟩ and |2⟩. As shown in Fig. 4, Fig. 5, Fig. 6, Fig. 7 over the range 0–10(×100,fs), the z-component of the relaxation term, $dn_{z}(t)/dt\propto {\left[\overset{↔}{\eta }(t)\cdot {\vec{n}}^{\left(av\right)}(t)(t)\right]}_{z}$, is primarily responsible for driving the state transition. The relaxation term towards the steady state is associated with fluctuations in this precession term, which depend on the coupling strength ($g_{k}$ or $\left|\Delta g_{k}\right|$): weak coupling leads to slower transitions, whereas stronger coupling accelerates the process. Furthermore, the effect of external magnetic field, defined by Eq. (5), enhances both the amplitude and fluctuation of the precession term, as evident in Fig. 6, Fig. 7, thereby amplifying the rate of quantum-state transitions.

A statistical interpretation of the stochastic dynamics can be obtained from the weighting functional and the width of the corridor path. The weighting functional (Fig. 8) quantifies the relative contribution of individual quantum trajectories, effectively describing the probability distribution of dynamical paths within the ensemble. Under weak system–bath coupling, the distribution is broad, signifying that numerous trajectories collectively sustain coherent evolution. As the coupling strength increases, the distribution narrows sharply, indicating the suppression of rare trajectories and the predominance of strongly damped paths. This behaviour highlights the environmental constraint on the system’s accessible dynamical space. The corridor width, in turn, measures the statistical spread of trajectories about the mean evolution. A wider corridor corresponds to greater fluctuations and enhanced sensitivity to external driving, whereas a narrower one reflects stronger decoherence and restricted dynamical variability. Fig. 10, Fig. 11 summarise these effects, showing how population-transfer efficiency—represented by the Bloch vector component $\vec{n}(t)$—depends on coupling strength. Narrowing of the corridor under strong coupling limits coherent transfer, while moderate coupling allows the external field to sustain coherence within the system.

## 5 Conclusions

In this work, we examine how system–bath coupling and external driving fields on tunnelling term jointly influence quantum-state transitions in the spin–boson model. This model serves as a fundamental framework for describing biological systems, chemical reaction mechanisms and quantum information. By comparing three complementary formalisms—the Redfield master equation, the HEOM, and the stochastic Langevin–Itô equation—we obtain trajectory-based descriptions of open-system dynamics. Our results show that the effectiveness of external driving depends sensitively on the coupling regime: in the weak- and intermediate-coupling limits, driving helps sustain coherence and promotes state transfer, whereas strong coupling suppresses coherence and accelerates the transition process.

Beyond the mean behaviour, the weighting functional and corridor width offer a statistical view of how stochastic fluctuations shape quantum trajectories, indicating how environmental noise restricts the accessible dynamical space. These results provide a coherent framework for analysing both average and fluctuation-driven features of quantum dynamics under external driving. The approach clarifies key aspects of dissipative two-level systems and highlights potential strategies for coherence control in quantum technologies, quantum picture in biological system and energy-transfer applications.

## CRediT authorship contribution statement

**Teerapat Uthailiang:** Writing – original draft, Software, Funding acquisition, Formal analysis. **Purin Issarakul:** Software, Formal analysis. **S. Boonchui:** Writing – review & editing, Project administration, Methodology, Formal analysis.

## Declaration of competing interest

The authors declare that they have no known competing financial interests or personal relationships that could have appeared to influence the work reported in this paper.

## Data Availability

The data sets used and analysed during the current study are available from the corresponding author (S. Boonchui) upon reasonable request.
