# Supplementary material for: Trajectory-based computational analysis of the quantum–classical transition in asymmetrically coupled spin–boson models
Source: Comput Struct Biotechnol J. 2026 Jan 16;34:1–14. doi: 10.1016/j.csbj.2026.01.006 (PMC12859464; doi:10.1016/j.csbj.2026.01.006)
Supplement: Multimedia Component 1 [file mmc1.pdf]

Supplementary Note: Trajectory-Based Computational Analysis of the Quantum–Classical Transition in Asymmetrically Coupled Spin–Boson Models

Teerapat Uthailiang[1], Purin Issarakul[1] & S. Boonchui[1,2,*]
[1] Department of Physics, Faculty of Science, Kasetsart University, Bangkok, 10900, Thailand
[2] Special Research Incubator Unit (SRIU), Faculty of Science, Kasetsart University, Bangkok, 10900, Thailand

Supplementary Note 1: The diagonalised system Hamiltonian and the relation between eigenstate $(|\pm\rangle)$ and state $|1\rangle, |2\rangle$

Since the system Hamiltonian $\hat{\mathbf{H}}_S$ following Eq. (3) of the manuscript is non-diagonal form, it can be transformed into a diagonal form as

$$\hat{\mathbf{H}}_S = \frac{\hbar\Omega}{2}\hat{\sigma}'_z, \qquad \text{where} \qquad \Omega = \sqrt{\epsilon^2 + \Delta^2}. \tag{S1.1}$$

This Hamiltonian satisfies $\hat{\mathbf{H}}_S|\pm\rangle = \pm(\Omega/2)|\pm\rangle$. Because this work focuses on the transition dynamics between the states $|1\rangle$ and $|2\rangle$, all analyses are performed in the eigenbasis $|\pm\rangle$ of the diagonalised Hamiltonian, and the results are later transformed back to the $|1\rangle, |2\rangle$ basis. The eigenstates are defined as

$$|+\rangle = \cos\frac{\theta}{2}|1\rangle + \sin\frac{\theta}{2}|2\rangle, \quad \text{and} \quad |-\rangle = -\sin\frac{\theta}{2}|1\rangle + \cos\frac{\theta}{2}|2\rangle \tag{S1.2}$$

with $\tan\theta = \Delta/\epsilon$. The Pauli operators in the eigenbasis are

$$\hat{\sigma}'_+ = |+\rangle\langle-|, \quad \hat{\sigma}'_- = |-\rangle\langle+|, \quad \text{and} \quad \hat{\sigma}'_z = |+\rangle\langle+| - |-\rangle\langle-|. \tag{S1.3}$$

Substituting the above eigenstate relations yields

$$\begin{aligned}
\hat{\sigma}'_+ &= \cos^2\frac{\theta}{2}|1\rangle\langle2| - \sin^2\frac{\theta}{2}|2\rangle\langle1| - \sin\frac{\theta}{2}\cos\frac{\theta}{2}(|1\rangle\langle1| - |2\rangle\langle2|),\\
\hat{\sigma}'_- &= -\sin^2\frac{\theta}{2}|1\rangle\langle2| + \cos^2\frac{\theta}{2}|2\rangle\langle1| - \sin\frac{\theta}{2}\cos\frac{\theta}{2}(|1\rangle\langle1| - |2\rangle\langle2|),\\
\hat{\sigma}'_z &= (\sin\theta)|1\rangle\langle2| + (\sin\theta)|2\rangle\langle1| + (\cos\theta)(|1\rangle\langle1| - |2\rangle\langle2|),
\end{aligned} \tag{S1.4}$$

Finally, in the $|1\rangle, |2\rangle$ representation, the Pauli operators are

$$\hat{\sigma}_+ = |1\rangle\langle2|, \quad \hat{\sigma}_- = |2\rangle\langle1|, \quad \text{and} \quad \hat{\sigma}_z = |1\rangle\langle1| - |2\rangle\langle2|. \tag{S1.5}$$

Corresponding author(s): S. Boonchui, fscistb@ku.ac.th

Similarly, in the eigenbasis $|\pm\rangle$, the Pauli operators are defined as $\hat{\sigma}_\pm = \frac{1}{2}(\hat{\sigma}_x \pm i\hat{\sigma}_y)$ within the $|1\rangle, |2\rangle$ representation. By applying the trigonometric identities $\cos^2\phi - \sin^2\phi = \cos(2\phi)$ and $2\sin\phi\cos\phi = \sin(2\phi)$, Eq. (S1.4) can be reformulated as

$$\begin{bmatrix} \hat{\sigma}'_x \\ \hat{\sigma}'_y \\ \hat{\sigma}'_z \end{bmatrix}_{\{|+\rangle,|-\rangle\}} = \begin{bmatrix} \cos\theta & 0 & -\sin\theta \\ 0 & 1 & 0 \\ \sin\theta & 0 & \cos\theta \end{bmatrix} \begin{bmatrix} \hat{\sigma}_x \\ \hat{\sigma}_y \\ \hat{\sigma}_z \end{bmatrix}_{\{|1\rangle,|2\rangle\}} \tag{S1.6}$$

Equivalently [Uthailiang et al. (2025)], this transformation can be written as

$$\hat{\sigma}'_\alpha = \sum_\eta \mathbf{T}_{\alpha\eta}\hat{\sigma}_\eta, \tag{S1.7}$$

and the operators $\hat{\sigma}'_\mu$ can be represented in the (x, y, z) basis as

$$\hat{\sigma}'_\mu = \sum_\alpha \mathbf{S}'_{\mu\alpha}\hat{\sigma}'_\alpha, \tag{S1.8}$$

where $\mathbf{S}'_{\mu\alpha}$ is the matrix-transformation element,

$$\mathbf{S}' = \begin{bmatrix} \frac{1}{2} & \frac{i}{2} & 0 \\ \frac{1}{2} & -\frac{i}{2} & 0 \\ 0 & 0 & 1 \end{bmatrix}. \tag{S1.9}$$

Substituting Eq. (S1.7) into Eq. (S1.8) gives

$$\hat{\sigma}'_\mu = \sum_{\alpha,\eta} \mathbf{S}'_{\mu\alpha}\mathbf{T}_{\alpha\eta}\hat{\sigma}_\eta \quad \text{or} \quad \hat{\sigma}'_\mu = \sum_\eta \mathbf{S}_{\mu\eta}\hat{\sigma}_\eta. \tag{S1.10}$$

where $\mathbf{S}_{\mu\eta} = \sum_\alpha \mathbf{S}'_{\mu\alpha}\mathbf{T}_{\alpha\eta}$ and

$$\mathbf{S} = \begin{bmatrix} \frac{1}{2} & \frac{i}{2} & 0 \\ \frac{1}{2} & -\frac{i}{2} & 0 \\ 0 & 0 & 1 \end{bmatrix} \begin{bmatrix} \cos\theta & 0 & -\sin\theta \\ 0 & 1 & 0 \\ \sin\theta & 0 & \cos\theta \end{bmatrix} = \begin{bmatrix} \frac{1}{2}\cos\theta & \frac{i}{2} & -\frac{1}{2}\sin\theta \\ \frac{1}{2}\cos\theta & -\frac{i}{2} & -\frac{1}{2}\sin\theta \\ \sin\theta & 0 & \cos\theta \end{bmatrix}. \tag{S1.11}$$

Using the relation in the path integral representation $\mathbf{Tr}\big(\hat{\sigma}'_\alpha\hat{\rho}(t)\big) = n'_\alpha(t)$, and then we can change the dynamics in the basis of eigenstates $|\pm\rangle$ into the basis of states $\{|1\rangle, |2\rangle\}$ as

$$\begin{bmatrix} n'_x(t) \\ n'_y(t) \\ n'_z(t) \end{bmatrix}_{\{|+\rangle,|-\rangle\}} = \begin{bmatrix} \cos\theta & 0 & -\sin\theta \\ 0 & 1 & 0 \\ \sin\theta & 0 & \cos\theta \end{bmatrix} \begin{bmatrix} n_x(t) \\ n_y(t) \\ n_z(t) \end{bmatrix}_{\{|1\rangle,|2\rangle\}} \tag{S1.12}$$

Supplementary Note 2: The Redfield master equation and the stochastic Langevin-Itô equation with the system operator $\hat{\sigma}_x$

Starting from the total Hamiltonian in Eq. (1) of the manuscript, the system–bath interaction term, $\hat{\mathbf{H}}_{\text{SB}}$, is defined using the system operator $\hat{L} = \hat{\sigma}_x$ as

$$\hat{\mathbf{H}}_{\text{SB}} = \hat{\sigma}_x \sum_k \hbar g_k(\hat{b}_k + \hat{b}_k^\dagger), \tag{S2.1}$$

which, in the eigenbasis $|\pm\rangle$, becomes

$$\hat{\mathbf{H}}_{\text{SB}} = (\sin\theta \cdot \hat{\sigma}'_z + \cos\theta \cdot \hat{\sigma}'_x) \sum_k \hbar g_k (\hat{b}_k + \hat{b}_k^\dagger). \tag{S2.2}$$

The corresponding Redfield master equation is expressed as

$$\frac{d}{dt}\hat{\rho}(t) = -\frac{i}{\hbar}[\hat{\mathbf{H}}_{\text{S}}, \hat{\rho}(t)] + \sum_{\mu=\pm,z} \Big(\Gamma_\mu(\omega, t)(\hat{\sigma}'_\mu \hat{\rho}(t) \hat{\sigma}'^\dagger_\mu - \hat{\sigma}'^\dagger_\mu \hat{\sigma}'_\mu \hat{\rho}(t)) + \text{h.c.} \Big), \tag{S2.3}$$

where $\hat{\mathbf{H}}_{\text{S}}$ follows eigenstate $|\pm\rangle$ form, $\mu = \pm, z$. The dissipation coefficients $\Gamma_\mu(\omega, t)$ are defined as

$$\begin{aligned} \Gamma_+(\Omega, t) &= \frac{1}{\hbar^2} \int_0^t d\tau\, e^{i\Omega\tau} C(\tau), \\ \Gamma_-(-\Omega, t) &= \frac{1}{\hbar^2} \int_0^t d\tau\, e^{-i\Omega\tau} C^*(\tau), \\ \Gamma_z(0, t) &= \frac{1}{\hbar^2} \int_0^t d\tau\, C(\tau), \end{aligned} \tag{S2.4}$$

where $\Gamma_\mu(\omega, t) = \frac{1}{2}\gamma_\mu(\omega, t) + iS_\mu(\omega, t)$ and the stochastic Langevin-Itô (stochastic Schrödinger) equation then reads

$$\begin{aligned} i\hbar \frac{d}{dt}|\varphi_\xi(t)\rangle = \hat{\mathbf{H}}_{\text{S}}|\varphi_\xi(t)\rangle &+ i\hbar \sum_{\mu=\pm,z} \gamma_\mu(\omega, t) \Big(2\langle\hat{\sigma}'_\mu\rangle \hat{\sigma}'^\dagger_\mu - \hat{\sigma}'_\mu \hat{\sigma}'^\dagger_\mu - \langle\hat{\sigma}'^\dagger_\mu\rangle\langle\hat{\sigma}'_\mu\rangle \Big) |\varphi_\xi(t)\rangle \\ &+ i\hbar \sum_{\mu=\pm,z} \sqrt{\gamma_\mu(\omega, t)} (\hat{\sigma}'^\dagger_\mu - \langle\hat{\sigma}'^\dagger_\mu\rangle) |\varphi_\xi(t)\rangle \dot{\xi}_\mu. \end{aligned} \tag{S2.5}$$

Supplementary Note 3: The Redfield master equation and the stochastic Langevin-Itô equation with the system operator $\sum_{m=1,2} \hat{\sigma}^+_m \hat{\sigma}^-_m$

Consider the Hamiltonian of the system-bath coupling, $\hat{\mathbf{H}}_{\text{SB}}$, where the system operator is given by $\hat{L} = \sum_{m=1,2} \hat{\sigma}^+_m \hat{\sigma}^-_m$ as

$$\hat{\mathbf{H}}_{\text{SB}} = \sum_{m=1,2} \hat{\sigma}^+_m \hat{\sigma}^-_m \sum_k \hbar g_k^{(m)} (\hat{b}_k + \hat{b}_k^\dagger), \tag{S3.1}$$

or in the eigenstate $|\pm\rangle$ basis form and set $g_k^{(2)} = \mathtt{r} g_k^{(1)}$ with $\mathtt{r}$ is the coupling strength ratio, following equation

$$\hat{\mathbf{H}}_{\text{SB}} = (\mathcal{R}'_z \hat{\sigma}'_z + \mathcal{R}'_x \hat{\sigma}'_x) \sum_k \hbar g_k^{(1)} (\hat{b}_k + \hat{b}_k^\dagger), \tag{S3.2}$$

where $\mathcal{R}'_x = (\mathtt{r} - 1)\sin\theta$, and $\mathcal{R}'_z = (1 - \mathtt{r})\cos\theta$. Then, the Redfield master equation has the same form following Eq. (S2.3), and the dissipation coefficient,

$\Gamma_\mu(\omega,t)$, for this case defined as

$$\begin{aligned}\Gamma_+(\Omega,t) &= \frac{|1-\mathtt{r}|}{2\hbar^2}\int_0^t d\tau\, e^{i\Omega\tau}C(\tau),\\ \Gamma_-(-\Omega,t) &= \frac{|1-\mathtt{r}|}{2\hbar^2}\int_0^t d\tau\, e^{-i\Omega\tau}C^*(\tau),\\ \Gamma_z(0,t) &= \frac{|1-\mathtt{r}|}{2\hbar^2}\int_0^t d\tau\, C(\tau),\end{aligned} \tag{S3.3}$$

Since the Redfield master equation of the same form, we have the following Eq. (S2.3), yields a stochastic Langevin-Itô equation of the same form as shown in Eq. (S2.5), differing only in the dissipation coefficient, $\Gamma_\mu(\omega,t)$. The study process, analysis, and definition of various variables for this case will be similar to the case $\hat{L}=\hat{\sigma}_x$.

Supplementary Note 4: The state-transition dynamics with the equation of motion for corridor path $\vec{\mathbf{n}}^{(av)}(t)$

The results shown in Figs. 4–7 of the manuscript are obtained by evaluating the individual components of the equation of motion. In this analysis, the Hamiltonian expressed in its diagonal representation, as given in Eq. (S1.1), can be written as

$$\hat{\mathbf{H}}_\mathrm{S} = \frac{\hbar\Omega}{2}\hat{k}'\cdot\hat{\vec{\sigma}}', \quad \text{with} \quad \vec{\mathbf{H}}_\mathrm{S} = \frac{\hbar\Omega}{2}\hat{k}' \tag{S4.1}$$

and the operator $\hat{\sigma}'_\pm$ is

$$\hat{\sigma}'_\pm = \frac{1}{2}(\hat{i}'\pm i\hat{j}')\cdot\hat{\vec{\sigma}}', \tag{S4.2}$$

where $\hat{\vec{\sigma}}' = \hat{\sigma}'_x\hat{i}' + \hat{\sigma}'_y\hat{j}' + \hat{\sigma}'_z\hat{k}'$, and the master equation, following Eq. (S2.3), is similar to Eq. (7) of the manuscript. So, the characteristic of the component in the equation of motion Eq. (14), in eigenstate $|\pm\rangle$ basis that is

$$\vec{\mathcal{B}}(t)\times\vec{\mathbf{n}}'^{(av)}(t) = \frac{2}{\hbar}\vec{\mathbf{H}}_\mathrm{S}\times\vec{\mathbf{n}}'^{(av)}(t) = -\Omega(n'_y\hat{i}' - n'_x\hat{j}'), \quad \text{where} \quad L^0_\mu = \sigma^0_\mu = 0, \tag{S4.3}$$

$$\begin{aligned}\overleftrightarrow{\eta}(t)\cdot\vec{\mathbf{n}}'^{(av)}(t) = &\Big(-[4\gamma_z(0,t)+2\gamma_+(\Omega,t)]n'_x - 2S_+(\Omega,t)n'_y\Big)\hat{i}'\\ &+\Big(-[4\gamma_z(0,t)+2\gamma_+(\Omega,t)]n'_y + 2S_+(\Omega,t)n'_x\Big)\hat{j}'\\ &+\Big(-4\gamma_+(\Omega,t)n'_z\Big)\hat{k}'\\ = &\,N'_x(t)\hat{i}' + N'_y(t)\hat{j}' + N'_z(t)\hat{k}',\end{aligned} \tag{S4.4}$$

and $\vec{\mathcal{T}}(t)=0$ which follows from the antisymmetric property of the vector (cross) product and symmetry of Pauli operators.

However, we can write Eqs. (S4.3)-(S4.4) on the vectors corresponding to the states $|1\rangle,|2\rangle$ using the relationship in Eq. (S1.12) to obtain:

$$\vec{\mathcal{B}}(t) \times \vec{\mathbf{n}}^{(av)}(t) = -\Omega\Big(n_y \cos\theta \hat{i} + (n_z \sin\theta - n_x \cos\theta)\hat{j} - n_y \sin\theta \hat{k}\Big), \tag{S4.5}$$

$$\overleftrightarrow{\eta}(t) \cdot \vec{\mathbf{n}}^{(av)}(t) = (N_x'(t)\cos\theta + N_z'(t)\sin\theta)\,\hat{i} + N_y'(t)\,\hat{j} + (N_z'(t)\cos\theta - N_x'(t)\sin\theta)\,\hat{k}, \tag{S4.6}$$

Supplementary Note 5: The equation of motion for stochastic path $\vec{\mathbf{n}}_\xi(t)$

If the stochastic density operator is defined as $\hat{\rho}_\xi(t) = |\varphi_\xi(t)\rangle\langle\varphi_\xi(t)|$, then differentiating with respect to time yields

$$\begin{aligned}\frac{d}{dt}(|\varphi_\xi(t)\rangle\langle\varphi_\xi(t)|) = \Big(\frac{d}{dt}|\varphi_\xi(t)\rangle\Big)\langle\varphi_\xi(t)| + |\varphi_\xi(t)\rangle\Big(\frac{d}{dt}\langle\varphi_\xi(t)|\Big) \\ +\Big(\frac{d}{dt}|\varphi_\xi(t)\rangle\Big)\Big(\frac{d}{dt}\langle\varphi_\xi(t)|\Big)dt.\end{aligned} \tag{S5.1}$$

By substituting the stochastic Langevin–Itô equation Eq. (26), as shown in the manuscript, into Eq. (S5.1), and noting that the system Hamiltonian satisfies $\hat{\mathbf{H}}_\mathrm{S}^\dagger = \hat{\mathbf{H}}_\mathrm{S}$, we obtain

$$\begin{aligned}\frac{d}{dt}\hat{\rho}_\xi(t) = -\frac{i}{\hbar}(\hat{\mathcal{H}}\hat{\rho}_\xi(t) - \hat{\rho}_\xi(t)\hat{\mathcal{H}}) + \sum_\mu \Big(\hat{\mathbf{L}}_\mu\hat{\rho}_\xi\hat{\mathbf{L}}_\mu^\dagger\dot{\xi}_\mu^*\dot{\xi}_\mu dt - \{\hat{\mathbf{L}}_\mu^\dagger\hat{\mathbf{L}}_\mu, \hat{\rho}_\xi\}\Big) \\ +\sum_\mu \hat{\mathbf{A}}_\mu + \hat{\mathbf{B}}_\mu\dot{\xi}_\mu dt + \hat{\mathbf{B}}_\mu^\dagger\dot{\xi}_\mu^* dt + \hat{\mathbf{C}}_\mu\dot{\xi}_\mu^*\dot{\xi}_\mu dt,\end{aligned} \tag{S5.2}$$

where the Hamiltonian $\hat{\mathcal{H}}$ and $\hat{\mathcal{H}}^\dagger$ are

$$\begin{aligned}\hat{\mathcal{H}} &= \hat{\mathbf{H}}_\mathrm{S} - i\hbar\langle\hat{\mathbf{L}}_\mu^\dagger\rangle\langle\hat{\mathbf{L}}_\mu\rangle - dt\,\hat{\mathbf{H}}_\mathrm{S}\Big(\langle\hat{\mathbf{L}}_\mu^\dagger\rangle\langle\hat{\mathbf{L}}_\mu\rangle + \langle\hat{\mathbf{L}}_\mu^\dagger\rangle\dot{\xi}_\mu^*\Big), \\ \hat{\mathcal{H}}^\dagger &= \hat{\mathbf{H}}_\mathrm{S} + i\hbar\langle\hat{\mathbf{L}}_\mu^\dagger\rangle\langle\hat{\mathbf{L}}_\mu\rangle - dt\,\hat{\mathbf{H}}_\mathrm{S}\Big(\langle\hat{\mathbf{L}}_\mu^\dagger\rangle\langle\hat{\mathbf{L}}_\mu\rangle + \langle\hat{\mathbf{L}}_\mu\rangle\dot{\xi}_\mu\Big),\end{aligned} \tag{S5.3}$$

and the function operators $\hat{\mathbf{A}}_\mu$, $\hat{\mathbf{B}}_\mu$, $\hat{\mathbf{B}^\dagger}_\mu$ and $\hat{\mathbf{C}}_\mu$ are defined as

$$\begin{aligned}\hat{\mathbf{A}}_\mu = 2\Big(\langle\hat{\mathbf{L}}_\mu^\dagger\rangle\hat{\mathbf{L}}_\mu\hat{\rho}_\xi + \hat{\rho}_\xi\hat{\mathbf{L}}_\mu^\dagger\langle\hat{\mathbf{L}}_\mu\rangle\Big) + dt\Big[\frac{1}{\hbar^2}\hat{\mathbf{H}}_\mathrm{S}\hat{\rho}_\xi\hat{\mathbf{H}}_\mathrm{S} + (2\langle\hat{\mathbf{L}}_\mu^\dagger\rangle\hat{\mathbf{L}}_\mu - \hat{\mathbf{L}}_\mu^\dagger\hat{\mathbf{L}}_\mu - \langle\hat{\mathbf{L}}_\mu^\dagger\rangle\langle\hat{\mathbf{L}}_\mu\rangle)\hat{\rho}_\xi \\ \times(2\hat{\mathbf{L}}_\mu^\dagger\langle\hat{\mathbf{L}}_\mu\rangle - \hat{\mathbf{L}}_\mu^\dagger\hat{\mathbf{L}}_\mu - \langle\hat{\mathbf{L}}_\mu^\dagger\rangle\langle\hat{\mathbf{L}}_\mu\rangle) + \frac{i}{\hbar}\Big((2\langle\hat{\mathbf{L}}_\mu^\dagger\rangle\hat{\mathbf{L}}_\mu - \hat{\mathbf{L}}_\mu^\dagger\hat{\mathbf{L}}_\mu)\hat{\rho}_\xi\hat{\mathbf{H}}_\mathrm{S} - \hat{\mathbf{H}}_\mathrm{S}\hat{\rho}_\xi(2\hat{\mathbf{L}}_\mu^\dagger\langle\hat{\mathbf{L}}_\mu\rangle - \hat{\mathbf{L}}_\mu^\dagger\hat{\mathbf{L}}_\mu)\Big)\Big],\end{aligned} \tag{S5.4}$$

$$\hat{\mathbf{B}}_\mu = (\hat{\mathbf{L}}_\mu - \langle\hat{\mathbf{L}}_\mu\rangle)\hat{\rho}_\xi + \frac{i}{\hbar}\hat{\mathbf{L}}_\mu\hat{\rho}_\xi\hat{\mathbf{H}}_\mathrm{S} + (\hat{\mathbf{L}}_\mu - \langle\hat{\mathbf{L}}_\mu\rangle)\hat{\rho}_\xi(2\hat{\mathbf{L}}_\mu^\dagger\langle\hat{\mathbf{L}}_\mu\rangle - \hat{\mathbf{L}}_\mu^\dagger\hat{\mathbf{L}}_\mu - \langle\hat{\mathbf{L}}_\mu^\dagger\rangle\langle\hat{\mathbf{L}}_\mu\rangle), \tag{S5.5}$$

$$\hat{\mathbf{B}}_\mu^\dagger = \hat{\rho}_\xi(\hat{\mathbf{L}}_\mu^\dagger - \langle\hat{\mathbf{L}}_\mu^\dagger\rangle) - \frac{i}{\hbar}\hat{\mathbf{H}}_\mathrm{S}\hat{\rho}_\xi\hat{\mathbf{L}}_\mu^\dagger + (2\langle\hat{\mathbf{L}}_\mu^\dagger\rangle\hat{\mathbf{L}}_\mu - \hat{\mathbf{L}}_\mu^\dagger\hat{\mathbf{L}}_\mu - \langle\hat{\mathbf{L}}_\mu^\dagger\rangle\langle\hat{\mathbf{L}}_\mu\rangle)\hat{\rho}_\xi(\hat{\mathbf{L}}_\mu^\dagger - \langle\hat{\mathbf{L}}_\mu^\dagger\rangle), \tag{S5.6}$$

and

$$\hat{\mathbf{C}}_\mu = \langle\hat{\mathbf{L}}_\mu^\dagger\rangle\langle\hat{\mathbf{L}}_\mu\rangle\hat{\rho}_\xi - \langle\hat{\mathbf{L}}_\mu^\dagger\rangle\hat{\mathbf{L}}_\mu\hat{\rho}_\xi - \hat{\rho}_\xi\hat{\mathbf{L}}_\mu^\dagger\langle\hat{\mathbf{L}}_\mu\rangle. \tag{S5.7}$$

However, since the infinitesimal time element satisfies $dt \ll 1$ and the ensemble averages of the first-order stochastic variables vanish, $\mathcal{M}(d\xi_\mu) = \mathcal{M}(d\xi_\mu^*) = 0$, Eq. (S5.2) can be simplified to the form

$$\begin{aligned}\frac{d}{dt}\hat{\rho}_\xi(t) = &-\frac{i}{\hbar}[\hat{\mathbf{H}}_{\mathrm{S}}, \hat{\rho}_\xi(t)] + \sum_\mu \Big(\hat{\mathbf{L}}_\mu \hat{\rho}_\xi(t)\hat{\mathbf{L}}_\mu^\dagger \dot{\xi}_\mu^* \dot{\xi}_\mu dt - \{\hat{\mathbf{L}}_\mu^\dagger \hat{\mathbf{L}}_\mu, \hat{\rho}_\xi(t)\}\Big) \\ &+ \sum_\mu \Big\{2\Big(1 - \frac{1}{2}\dot{\xi}_\mu^* \dot{\xi}_\mu dt\Big)\Big(\langle \hat{\mathbf{L}}_\mu^\dagger\rangle \hat{\mathbf{L}}_\mu \hat{\rho}_\xi(t) + \hat{\rho}_\xi(t)\hat{\mathbf{L}}_\mu^\dagger \langle \hat{\mathbf{L}}_\mu\rangle - \langle \hat{\mathbf{L}}_\mu^\dagger\rangle\langle \hat{\mathbf{L}}_\mu\rangle \hat{\rho}_\xi(t)\Big)\Big\},\end{aligned} \tag{S5.8}$$

and Eq. (S5.8) is expressed as

$$\frac{d}{dt}\hat{\rho}_\xi(t) = \frac{d}{dt}\rho_\xi^0(t)\mathbb{I} + \frac{d}{dt}\vec{\rho}_\xi(t)\cdot\hat{\vec{\sigma}}, \quad \text{with} \quad \hat{\rho}_\xi(t) = \rho_\xi^0(t)\hat{\mathbb{I}} + \vec{\rho}_\xi \cdot \hat{\vec{\sigma}}, \tag{S5.9}$$

then the corresponding components satisfy

$$\begin{aligned}\frac{d}{dt}\rho_\xi^0(t) = \sum_\mu \Big\{&2(1 - \frac{1}{2}\dot{\xi}_\mu^* \dot{\xi}_\mu dt)\Big[(\langle \hat{\mathbf{L}}_\mu^\dagger\rangle \mathbf{L}_\mu^0 + \langle \hat{\mathbf{L}}_\mu\rangle \mathbf{L}_\mu^{0\dagger} - \mathbf{L}_\mu^{0\dagger}\mathbf{L}_\mu^0 - \langle \hat{\mathbf{L}}_\mu^\dagger\rangle\langle \hat{\mathbf{L}}_\mu\rangle - \vec{\mathbf{L}}_\mu^\dagger \cdot \vec{\mathbf{L}}_\mu)\rho_\xi^0(t) \\ &+ \Big((\langle \hat{\mathbf{L}}_\mu^\dagger\rangle - \mathbf{L}_\mu^{0\dagger})\vec{\mathbf{L}}_\mu + (\langle \hat{\mathbf{L}}_\mu\rangle - \mathbf{L}_\mu^0)\vec{\mathbf{L}}_\mu^\dagger - i\vec{\mathbf{L}}_\mu^\dagger \times \vec{\mathbf{L}}_\mu\Big)\cdot \vec{\rho}_\xi(t)\Big]\Big\},\end{aligned} \tag{S5.10}$$

and

$$\frac{d}{dt}\vec{\rho}_\xi(t) = \vec{\mathcal{B}}_\xi(t) \times \vec{\rho}_\xi(t) + \overleftrightarrow{\eta}_\xi(t)\cdot\vec{\rho}_\xi(t) + \vec{\mathcal{T}}_\xi(t). \tag{S5.11}$$

Let the stochastic density vector be defined as $\vec{\rho}_\xi(t) = \frac{1}{2}\vec{\mathbf{n}}_\xi(t)$ and $\rho_\xi^0(t) = \frac{1}{2}\mathbb{I}$. Hence, Eq. (S5.11) can be rewritten in the form

$$\frac{d}{dt}\vec{\mathbf{n}}_\xi(t) = \vec{\mathcal{B}}_\xi(t) \times \vec{\mathbf{n}}_\xi(t) + \overleftrightarrow{\eta}_\xi(t)\cdot\vec{\mathbf{n}}_\xi(t) + \vec{\mathcal{T}}_\xi(t), \tag{S5.12}$$

so called the equation of motion for the stochastic path $\vec{\mathbf{n}}_\xi(t)$, where $\vec{\mathcal{B}}_\xi(t)$, $\overleftrightarrow{\eta}_\xi(t)\cdot\vec{\mathbf{n}}_\xi(t)$, and $\vec{\mathcal{T}}_\xi(t)$ are defined as

$$\begin{aligned}\vec{\mathcal{B}}_\xi(t) = \frac{2}{\hbar}\vec{\mathbf{H}}_{\mathrm{S}} + \sum_\mu \Big\{&i\dot{\xi}_\mu^* \dot{\xi}_\mu dt\Big((\mathbf{L}_\mu^{0\dagger}\vec{\mathbf{L}}_\mu - \mathbf{L}_\mu^0 \vec{\mathbf{L}}_\mu^\dagger) - \frac{i}{2}(\vec{\mathbf{L}}_\mu \times \vec{\mathbf{L}}_\mu^\dagger)\Big) \\ &+ 2i\Big(1 - \frac{1}{2}\dot{\xi}_\mu^* \dot{\xi}_\mu dt\Big)\Big(\langle \hat{\mathbf{L}}_\mu^\dagger\rangle \vec{\mathbf{L}}_\mu - \langle \hat{\mathbf{L}}_\mu\rangle \vec{\mathbf{L}}_\mu^\dagger\Big)\Big\},\end{aligned} \tag{S5.13}$$

$$\begin{aligned}\overleftrightarrow{\eta}_\xi(t)\cdot\vec{\mathbf{n}}_\xi(t) = \sum_\mu \Big\{&\dot{\xi}_\mu^* \dot{\xi}_\mu dt(\vec{\mathbf{n}}_\xi(t)\cdot\vec{\mathbf{L}}_\mu^\dagger)\vec{\mathbf{L}}_\mu - 2(\vec{\mathbf{L}}_\mu^\dagger \cdot \vec{\mathbf{L}}_\mu)\vec{\mathbf{n}}_\xi(t) \\ &+ 2(1 - \frac{1}{2}\dot{\xi}_\mu^* \dot{\xi}_\mu dt)(\langle \hat{\mathbf{L}}_\mu^\dagger\rangle \mathbf{L}_\mu^0 + \langle \hat{\mathbf{L}}_\mu\rangle \mathbf{L}_\mu^{0\dagger} + \langle \hat{\mathbf{L}}_\mu^\dagger\rangle\langle \hat{\mathbf{L}}_\mu\rangle - \mathbf{L}_\mu^{0\dagger}\mathbf{L}_\mu^0)\vec{\mathbf{n}}_\xi(t)\Big\},\end{aligned} \tag{S5.14}$$

$\vec{A} \times (\vec{B} \times \vec{C}) = (\vec{A}\cdot\vec{C})\vec{B} - (\vec{A}\cdot\vec{B})\vec{C}$, and

$$\begin{aligned}\vec{\mathcal{T}}_\xi(t) = \sum_\mu \Big\{&2i(1 + \frac{1}{2}\dot{\xi}_\mu^* \dot{\xi}_\mu dt)(\vec{\mathbf{L}}_\mu \times \rho_\xi^0(t)\vec{\mathbf{L}}_\mu^\dagger) \\ &+ 2(1 - \frac{1}{2}\dot{\xi}_\mu^* \dot{\xi}_\mu dt)[\langle \hat{\mathbf{L}}_\mu^\dagger\rangle - \mathbf{L}_\mu^{0\dagger})\rho_\xi^0(t)\vec{\mathbf{L}}_\mu + (\langle \hat{\mathbf{L}}_\mu\rangle - \mathbf{L}_\mu^0)\rho_\xi^0(t)\vec{\mathbf{L}}_\mu^\dagger]\Big\}.\end{aligned} \tag{S5.15}$$

However, If Eq. (S5.9) is averaged over the Wiener processes (ensemble average), Eq. (S5.10) is zero and Eq. (S5.11) becomes the equation of motion for corridor path (averaged path).

Supplementary Note 6: Component of Bloch vector representation

From the stochastic state described by Eq. (38) in the manuscript, and following the formulation in Refs. [Kirchner (2010); Feynman et al. (1957)], the expression can be written as

$$\begin{aligned}\hat{\rho}_\xi(t) = |\varphi_\xi(t)\rangle\langle\varphi_\xi(t)| &= \begin{bmatrix} |\alpha(t;\ell_\beta(t))|^2 & \alpha(t;\ell_\beta(t))\beta^*(t;\ell_\beta(t)) \\ \alpha^*(t;\ell_\beta(t))\beta(t;\ell_\beta(t)) & |\beta(t;\ell_\beta(t))|^2 \end{bmatrix} \\ &= \frac{1}{2}\Big(\overline{n}'_x(t;\ell_\beta(t))\hat{\sigma}'_x + \overline{n}'_y(t;\ell_\beta(t))\hat{\sigma}'_y + \overline{n}'_z(t;\ell_\beta(t))\hat{\sigma}'_z\Big) + c\mathbb{I},\end{aligned} \tag{S6.1}$$

where $\hat{\sigma}'_x$, $\hat{\sigma}'_y$, and $\hat{\sigma}'_z$ denote the Pauli operators expressed in the eigenbasis $|\pm\rangle$ basis, $\mathbb{I}$ is the identity operator and c is a constant. The components $\overline{n}'_x(t;\ell_\beta(t))$, $\overline{n}'_y(t;\ell_\beta(t))$, $\overline{n}'_z(t;\ell_\beta(t))$ are related to $\alpha(t;\ell_\beta(t))$ and $\beta(t;\ell_\beta(t))$ through the following expressions:

$$\begin{aligned}\hat{\rho}_\xi(t) &= \frac{1}{2}\Big(\overline{n}'_x(t;\ell_\beta(t))\hat{\sigma}'_x + \overline{n}'_y(t;\ell_\beta(t))\hat{\sigma}'_y + \overline{n}'_z(t;\ell_\beta(t))\hat{\sigma}'_z\Big) + c\mathbb{I} \\ &= \begin{bmatrix} \frac{1}{2}\overline{n}'_z(t;\ell_\beta(t)) + c & \frac{1}{2}\Big(\overline{n}'_x(t;\ell_\beta(t)) - i\overline{n}'_y(t;\ell_\beta(t))\Big) \\ \frac{1}{2}\Big(\overline{n}'_x(t;\ell_\beta(t)) + i\overline{n}'_y(t;\ell_\beta(t))\Big) & -\frac{1}{2}\overline{n}'_z(t;\ell_\beta(t)) + c \end{bmatrix}.\end{aligned} \tag{S6.2}$$

Thus, we obtain

$$\begin{aligned}&\begin{bmatrix} \frac{1}{2}\overline{n}'_z(t;\ell_\beta(t)) + c & \frac{1}{2}\Big(\overline{n}'_x(t;\ell_\beta(t)) - i\overline{n}'_y(t;\ell_\beta(t))\Big) \\ \frac{1}{2}\Big(\overline{n}'_x(t;\ell_\beta(t)) + i\overline{n}'_y(t;\ell_\beta(t))\Big) & -\frac{1}{2}\overline{n}'_z(t;\ell_\beta(t)) + c \end{bmatrix} \\ &\qquad = \begin{bmatrix} |\alpha(t;\ell_\beta(t))|^2 & \alpha(t;\ell_\beta(t))\beta^*(t;\ell_\beta(t)) \\ \alpha^*(t;\ell_\beta(t))\beta(t;\ell_\beta(t)) & |\beta(t;\ell_\beta(t))|^2 \end{bmatrix},\end{aligned} \tag{S6.3}$$

when we examine each component, we derive the following equation:

$$|\alpha(t;\ell_\beta(t))|^2 = \frac{1}{2}\overline{n}'_z(t;\ell_\beta(t)) + c, \tag{S6.4}$$

$$|\beta(t;\ell_\beta(t))|^2 = -\frac{1}{2}\overline{n}'_z(t;\ell_\beta(t)) + c, \tag{S6.5}$$

$$\alpha(t;\ell_\beta(t))\beta^*(t;\ell_\beta(t)) = \frac{1}{2}\Big(\overline{n}'_x(t;\ell_\beta(t)) - i\overline{n}'_y(t;\ell_\beta(t))\Big), \tag{S6.6}$$

$$\alpha^*(t;\ell_\beta(t))\beta(t;\ell_\beta(t)) = \frac{1}{2}\Big(\overline{n}'_x(t;\ell_\beta(t)) + i\overline{n}'_y(t;\ell_\beta(t))\Big), \tag{S6.7}$$

and upon solving the aforementioned equation, we find that [Feynman et al. (1957)]

$$\overline{n}'_x(t;\ell_\mu(t)) = 2\,\mathrm{Re}[\alpha^*(t;\ell_\mu(t))\beta(t;\ell_\mu(t))], \tag{S6.8}$$

$$\overline{n}'_y(t;\ell_\mu(t)) = 2\,\mathrm{Im}[\alpha^*(t;\ell_\mu(t))\beta(t;\ell_\mu(t))], \tag{S6.9}$$

$$\overline{n}'_z(t;\ell_\mu(t)) = |\alpha(t;\ell_\mu(t))|^2 - |\beta(t;\ell_\mu(t))|^2. \tag{S6.10}$$

Alternatively, the density operator obtained from either the master equation or the HEOM method can be expressed in matrix form as

$$\hat{\rho}(t) = \begin{bmatrix} \rho_{11}(t) & \rho_{12}(t) \\ \rho_{21}(t) & \rho_{22}(t) \end{bmatrix}, \tag{S6.11}$$

where the values of $\rho_{11}(t)$ and $\rho_{22}(t)$ are the real number, and $\rho_{12}(t)$ and $\rho_{21}(t)$ are the complex number with $\rho_{21}(t) = \rho_{12}^*(t)$. Then, Eq. (S6.11) compare form Eq. (S6.1) will get the relation of component in the Bloch vector in states $|1\rangle, |2\rangle$ basis as

$$\overline{n}_x(t) = 2Re[\rho_{12}(t)], \tag{S6.12}$$

$$\overline{n}_y(t) = 2Im[\rho_{21}(t)], \tag{S6.13}$$

$$\overline{n}_z(t) = \rho_{11}(t) - \rho_{22}(t). \tag{S6.14}$$

Next, we consider the solution obtained from the stochastic equation in the eigenstate basis, which can be expressed in the form Eq. [38] in manuscript, and then substitute Eq. (S1.2) for the state $|1\rangle, |2\rangle$ basis form, we obtain that

$$|\varphi_\xi(t)\rangle = \alpha(t;\ell_\beta(t))|+\rangle + \beta(t;\ell_\beta(t))|-\rangle = \overline{\alpha}(t;\ell_\beta(t))|1\rangle + \overline{\beta}(t;\ell_\beta(t))|2\rangle, \tag{S6.15}$$

where

$$\overline{\alpha}(t;\ell_\mu(t)) = \alpha(t;\ell_\mu(t))\cos\frac{\theta}{2} - \beta(t;\ell_\mu(t))\sin\frac{\theta}{2}, \tag{S6.16}$$

$$\overline{\beta}(t;\ell_\mu(t)) = \alpha(t;\ell_\mu(t))\sin\frac{\theta}{2} + \beta(t;\ell_\mu(t))\cos\frac{\theta}{2}. \tag{S6.17}$$

If we choose to define the density operator $\hat{\rho}_\xi(t)$ using Eq. (S6.15), we will obtain an equation similar to Eq. (S6.1), which is

$$\begin{aligned} \hat{\rho}_\xi(t) = |\varphi_\xi(t)\rangle\langle\varphi_\xi(t)| &= \begin{bmatrix} |\overline{\alpha}(t;\ell_\mu(t))|^2 & \overline{\alpha}(t;\ell_\mu(t))\overline{\beta}^*(t;\ell_\mu(t)) \\ \overline{\alpha}^*(t;\ell_\mu(t))\overline{\beta}(t;\ell_\mu(t)) & |\overline{\beta}(t;\ell_\mu(t))|^2 \end{bmatrix} \\ &= \frac{1}{2}\Big(\overline{n}_x(t;\ell_\mu(t))\hat{\sigma}_x + \overline{n}_y(t;\ell_\mu(t))\hat{\sigma}_y + \overline{n}_z(t;\ell_\mu(t))\hat{\sigma}_z\Big) + c\mathbb{I}, \end{aligned} \tag{S6.18}$$

where $\hat{\sigma}_x$, $\hat{\sigma}_y$, and $\hat{\sigma}_z$ are the Pauli operators in the $\{|1\rangle, |2\rangle\}$ basis and allows us to obtain $\overline{n}_x(t;\ell_\mu(t))$, $\overline{n}_y(t;\ell_\mu(t))$, and $\overline{n}_z(t;\ell_\mu(t))$ in the $\{|1\rangle, |2\rangle\}$ basis as well. It can be seen that we analyse through the eigenstates $|\pm\rangle$ basis. Once the results are obtained, we then transform them into the $\{|1\rangle, |2\rangle\}$ basis by using the relation given in Eq. (S1.6) afterward.

Supplementary Note 7: Additional Results

7.1 Compare three methods

In this work, we employ three approaches—the Redfield master equation, the hierarchical equations of motion (HEOM), and the stochastic Schrödinger equation

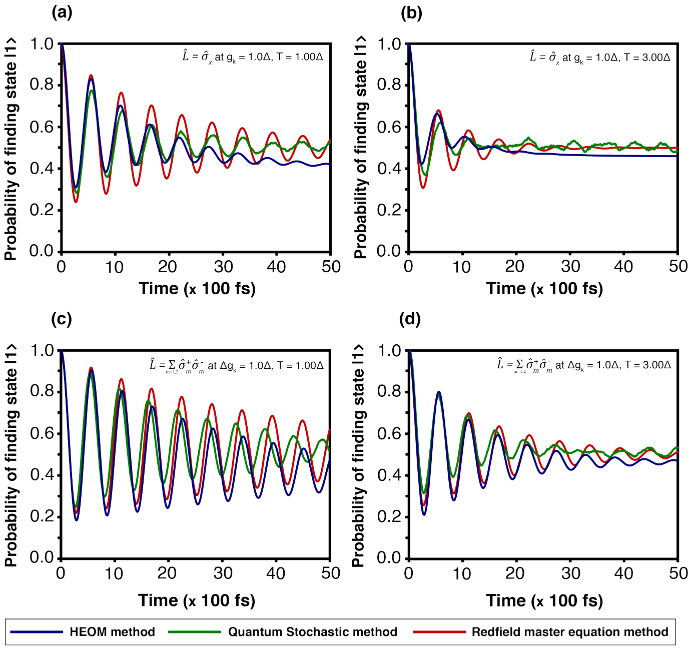

Figure 1: Comparison of the time-dependent probability of the state $|1\rangle$, calculated using the system Hamiltonian in Eq. [3], as shown in the manuscript, without an external magnetic field and assuming an under-damped Brownian spectral density. Results from the Redfield master equation (red), the stochastic Schrödinger equation (green), and HEOM (blue) are shown for: (**a**) $\hat{L} = \hat{\sigma}_x$ with $g_k = 1.0\Delta$ at $T = 1.00\Delta[76.45 \times 10^{-13}\text{K·s}]$; (**b**) same coupling at $T = 3.00\Delta[76.45 \times 10^{-13}\text{K·s}]$; (**c**) $\hat{L} = \sum_{m=1,2} \hat{\sigma}_m^+ \hat{\sigma}_m^-$ with $g_k^{(1)} = 2.0\Delta, g_k^{(2)} = 1.0\Delta$ at $T = 1.00\Delta[76.45 \times 10^{-13}\text{K·s}]$; (**d**) same local coupling at $T = 3.00\Delta[76.45 \times 10^{-13}\text{K·s}]$.

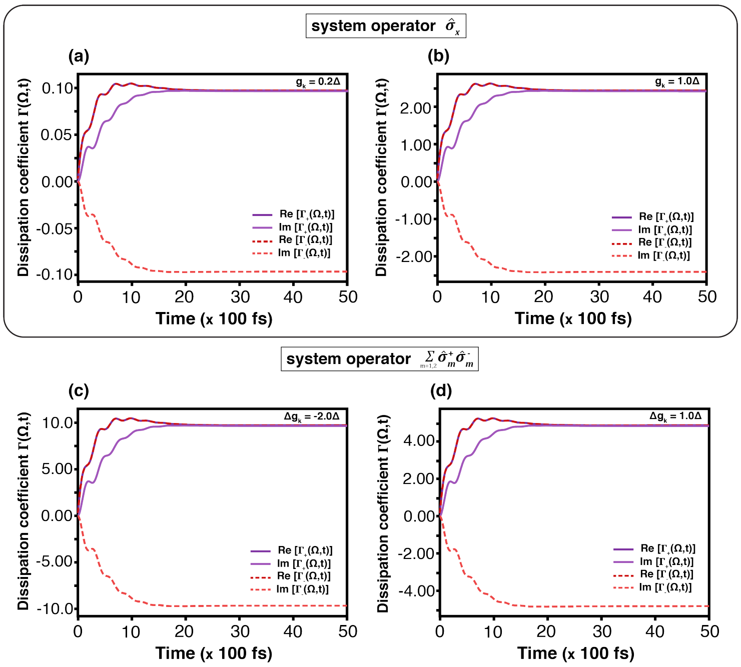

Figure 2: Time evolution of the dissipation coefficient $\Gamma_{\pm}(\Omega, t)$: (**a**) $\hat{L} = \hat{\sigma}_x$ with $g_k = 1.0\Delta$ at $T = 1.00\Delta[76.45 \times 10^{-13}\text{K·s}]$; (**b**) same coupling at $T = 3.00\Delta[76.45 \times 10^{-13}\text{K·s}]$; (**c**) $\hat{L} = \sum_{m=1,2} \hat{\sigma}_m^+ \hat{\sigma}_m^-$ with $g_k^{(1)} = 2.0\Delta, g_k^{(2)} = 1.0\Delta$ at $T = 1.00\Delta[76.45 \times 10^{-13}\text{K·s}]$; (**d**) same local coupling at $T = 3.00\Delta[76.45 \times 10^{-13}\text{K·s}]$.

(quantum state diffusion). Using the spectral-density parameters listed in Table 1, the population of state $|1\rangle$ obtained from all three methods shows close agreement at elevated temperatures. In particular, for $T = 3.00\Delta$, the results converge as shown in Fig. 1.

At strong coupling, the HEOM method provides a more accurate description of the rapid coherence decay, whereas the Redfield equation slightly underestimates the damping due to its perturbative character. The stochastic formulation reproduces the averaged dynamics with high fidelity, demonstrating its reliability as a complementary viewpoint alongside analytic and numerical approaches. Overall, the results confirm that the stochastic quantum-trajectory method remains consistent with established techniques across weak- to strong-coupling regimes, and is particularly valuable for analysing statistical properties of trajectories—such as the weighting functional and corridor width—that cannot be obtained from either Redfield or HEOM. Furthermore, the spin-boson model has a coupling between the system and the bath with the system operator $\sum_{m=1,2} \hat{\sigma}_m^+ \hat{\sigma}_m^-$, which is similar to the coupling in the photosynthetic system or others biological system, as mentioned in the manuscript, where the probability of finding the state $|1\rangle$ obtained from HEOM when the temperature is fixed depends on the relative coupling strength $|\Delta g_k|$, which is similar to the result in [Uthailiang et al. (2025)] which used a different approach.

Table 1: Parameters used in calculating the spectral density in the under-damped Brownian motion type, as described by Eq. (10) in the manuscript, and related parameters for the system.

**Parameters**	**Value [unit]**	**(non) SI unit**
Δ (the electronic coupling of state)	1.0 $[10^{13}\,\mathrm{s}^{-1}]$	53 cm^{-1}
ω_0 (the resonance frequency)	Δ $[1.055 \times 10^{-21}$ J.s$]$	53 cm^{-1}
ϵ (the energy of state)	0.5Δ $[1.055 \times 10^{-21}$ J.s$]$	26.5 cm^{-1}
γ (the width coefficient)	0.5Δ $[1.055 \times 10^{-21}$ J.s$]$	26.5 cm^{-1}
low temperature T	1.00$\Delta[76.45 \times 10^{-13}$ K.s$]$	77 K
high temperature T	3.00$\Delta[76.45 \times 10^{-13}$ K.s$]$	230 K

For the biological system, we consider the PC645 complex following Ref. [Blau et al. (2018)], where DBV$_c$ is assigned as the donor and PCB82$_c$ as the acceptor. The corresponding parameter set is specified in Table 2.

Table 2: Parameters in realistic case Refs. [Blau et al. (2018)] in unit of our work for the spectral density in the under-damped Brownian motion type, as described by Eq. (10) in the manuscript, and related parameters for the system.

**Parameters**	**Value [unit]**	**unit in our work**
Δ (fixed)	24.3 cm^{-1}	1.0Δ
ϵ	1600 cm^{-1}	65.84Δ
ω_0	1500 cm^{-1}	61.73Δ
γ	15 cm^{-1}	0.62Δ
Coupling strength of DBV$_c$	180 cm^{-1}	7.41Δ
Coupling strength of PCB82$_c$	500 cm^{-1}	20.58Δ
Temperature T	300 K	8.58Δ

7.2 Steady time (τ_{sd}) and Steady probability (P_{sd})

The steady time and steady probability reported in Fig. 2 of the manuscript are consistent with the numerical values listed in Table 2 for the system operator $\hat{\sigma}_x$ and in Table 4 for the operator $\sum_{m=1,2} \hat{\sigma}_m^+ \hat{\sigma}_m^-$, including the comparison across different bath temperatures. These results were obtained using the HEOM method, implemented following the numerical procedure of Ref., [Lambert et al. (2023)] for the spin–boson model. To identify the steady time and steady probability in the absence of an external magnetic field, we impose the criterion that the probabilities evaluated at time τ_{sd} and $t > \tau_{sd}$, differ by less than a threshold of order 10^{-8}. When this condition is met, τ_{sd} is identified as the steady time t_{sd}, and the corresponding probability is taken as the steady probability P_{sd}, as shown

$$|P(\tau_{sd}) - P(t)| < \mathcal{O}(10^{-8}). \tag{S7.1}$$

The values in Tables 3 and 4 result from numerical calculations performed with threshold values between 10^{-10} and 10^{-8}. Although the precise value of τ_{sd} may vary slightly with the chosen threshold, the overall conclusions remain unaffected.

However, as discussed in the section on steady time τ_{sd}, which characterises the lifetime of the probability dynamics, the steady time depends on the bath temperature T and the coupling strength λ through $\tau_{sd} \propto \lambda^{-2}\big(T_0(\omega)/T\big)$, where $T_0(\omega) = \hbar\omega/k_B$ is the Einstein temperature. Using the approximation $\coth(T_0(\omega)/2T) \approx 2T/T_0(\omega)$, this scaling follows from interpreting the dissipation coefficient $\Gamma_\mu(\Omega, t)$ as the factor governing probability decay. Under this interpretation, the dissipation coefficient behaves as an effective lifetime, i.e. $\tau \sim 1/\Gamma_\mu(\Omega, t)$.

7.3 The state-transition dynamics through the equation of motion for corridor path $\vec{\mathbf{n}}^{(av)}(t)$ (the Bloch equation)

In analysing the state-transition dynamics discussed in Section 3.2, we describe the evolution of the two-level system through its mapping onto the Bloch sphere, as given by Eq. [42] and shown in Fig. 3 in the manuscript. The resulting trajectory must satisfy the equation of motion for corridor path $\vec{\mathbf{n}}^{(av)}(t)$. Our analysis shows that the precession term, $\vec{\mathcal{B}}(t) \times \vec{\mathbf{n}}^{(av)}(t)$, plays the dominant role in driving the transition. This contribution depends on both the system Hamiltonian, $\vec{\mathbf{H}}_\mathrm{S}$, and the dissipation coefficients $\Gamma_\mu(\omega, t)$. During the initial transition phase, the influence of dissipation can be identified from Figs. 2, which display the coefficients $\Gamma_\pm(\Omega, t)$ for the two coupling operators, $\hat{\sigma}_x$ and $\sum_{m=1,2} \hat{\sigma}_m^+ \hat{\sigma}_m^-$, respectively.

Furthermore, we examine the equation of motion in an extreme limiting case. The first case corresponds to the bath-free scenario, in which both the relaxation and fluctuation terms vanish. The dynamics therefore reduce to pure precession determined solely by the system Hamiltonian $\vec{\mathbf{H}}_\mathrm{S}$. In this limit, the equation of motion takes the form

$$\frac{d}{dt}\vec{\mathbf{n}}(t) = \frac{2}{\hbar}\vec{\mathbf{H}}_\mathrm{S} \times \vec{\mathbf{n}}(t). \tag{S7.2}$$

Table 3: The steady time (τ_{sd}) and the steady probability (P_{sd}) in the case system operator $\hat{\sigma}_x$.

$\mathbf{g_k}$ [Δ]	T = 1.00Δ		T = 3.00Δ	
	$\tau_{\mathbf{sd}}$ [×100 fs]	$\mathbf{P_{sd}}$	$\tau_{\mathbf{sd}}$ [×100 fs]	$\mathbf{P_{sd}}$
0.1	NaN	NaN	3905.89	0.460
0.2	3382.74	0.387	1028.60	0.460
0.3	1635.36	0.387	993.60	0.459
0.4	1586.56	0.387	595.56	0.459
0.5	1060.11	0.387	401.04	0.459
0.6	764.08	0.387	290.53	0.459
0.7	579.26	0.387	222.02	0.459
0.8	456.85	0.387	176.02	0.459
0.9	370.44	0.388	143.51	0.459
1.0	307.23	0.388	120.51	0.459
1.1	260.03	0.388	103.01	0.459
1.2	223.22	0.389	90.01	0.459
1.3	194.42	0.389	80.01	0.459
1.4	165.52	0.389	72.51	0.459
1.5	148.01	0.390	66.51	0.459
1.6	133.01	0.390	63.01	0.459
1.7	121.01	0.390	61.01	0.459
1.8	111.01	0.391	60.01	0.459
1.9	103.01	0.391	60.51	0.459
2.0	96.01	0.391	62.01	0.459

The second case corresponds to the high-temperature limit, in which the bath correlation function can be approximated as

$$C(\tau) \approx \int_0^\infty d\omega\, J(\omega)\Big[\Big(\frac{2T}{T_0(\omega)}\Big)\cos(\omega\tau) - i\sin(\omega\tau)\Big], \quad \text{for} \quad T >> T_0. \tag{S7.3}$$

So, the dissipation coefficient $\Gamma_\pm(\Omega, t)$ in real and imaginary form for high temperature limit as shown

$$\text{Re}[\Gamma_\pm(\Omega,t)] = \frac{t}{2}\int_0^\infty d\omega'\, J(\omega')\frac{2T}{T_0(\omega')} - \frac{t^3}{12}\int_0^\infty d\omega'\, J(\omega')\frac{2T}{T_0(\omega')}(\Omega^2 + \omega'^2), \tag{S7.4}$$

$$\text{Im}[\Gamma_\pm(\Omega,t)] = \pm\frac{t^2}{4}\Big[\Omega\int_0^\infty d\omega'\, J(\omega')\frac{2T}{T_0(\omega')} - \int_0^\infty d\omega'\, J(\omega')\omega'\Big]. \tag{S7.5}$$

The real part $\text{Re}[\Gamma_\pm(\Omega,t)]$ increases linearly in time t and is proportional to the temperature T. Consequently, at higher temperatures the dissipation develops more rapidly, as the temperature-dependent coefficient in $\text{Re}[\Gamma_\pm(\Omega,t)]$ causes the corridor to narrow more quickly and enhances the relaxation term.

In the third case, corresponding to the extreme low-temperature regime, the factor $\coth(T_0(\omega)/2T)$ takes a value close to 1 and remains effectively constant

Table 4: The steady time (τ_{sd}) and the steady probability (P_{sd}) in the case system operator $\sum_{m=1,2}\hat{\sigma}_m^+\hat{\sigma}_m^-$ for example case $g_k^{(1)} > g_k^{(2)}$ where $g_k^{(1)} = 2.0\Delta$, $g_k^{(2)} = [0, 1.9]\Delta$.

Δg_k [Δ]	T = 1.00Δ		T = 3.00Δ	
	τ_{sd} [×100 fs]	P_{sd}	τ_{sd} [×100 fs]	P_{sd}
0.1	NaN	NaN	NaN	NaN
0.2	NaN	NaN	4420.94	0.460
0.3	4981.50	0.391	1623.16	0.460
0.4	2424.64	0.392	1381.14	0.461
0.5	1747.67	0.394	872.09	0.462
0.6	1517.85	0.396	480.55	0.463
0.7	993.10	0.397	480.55	0.462
0.8	847.58	0.398	339.53	0.463
0.9	687.97	0.399	296.03	0.463
1.0	587.76	0.400	228.52	0.464
1.1	423.34	0.400	205.02	0.464
1.2	360.34	0.401	176.52	0.464
1.3	297.63	0.402	151.02	0.465
1.4	254.73	0.403	139.01	0.465
1.5	228.62	0.403	119.51	0.465
1.6	189.02	0.403	110.51	0.465
1.7	141.61	0.404	89.01	0.465
1.8	132.61	0.404	78.01	0.465
1.9	123.61	0.404	77.01	0.465
2.0	106.81	0.404	64.01	0.465

for $0 < T \leq T_0$. Consequently, the bath correlation function reduces to the form

$$C(\tau) \approx \int_0^\infty d\omega J(\omega)\Big[\cos(\omega\tau) - i\sin(\omega\tau)\Big] = \int_0^\infty d\omega J(\omega)e^{-i\omega\tau}, \quad \text{for} \quad 0 < T \leq T_0. \tag{S7.6}$$

So, the dissipation coefficient $\Gamma_\pm(\Omega, t)$ in real and imaginary form for low temperature regime as shown

$$\text{Re}[\Gamma_\pm(\Omega,t)] = \frac{t}{2}\int_0^\infty d\omega'\, J(\omega') - \frac{t^3}{12}\int_0^\infty d\omega'\, J(\omega')(\Omega^2 + \omega'^2), \tag{S7.7}$$

$$\text{Im}[\Gamma_\pm(\Omega,t)] = \pm\frac{t^2}{4}\Big[\Omega\int_0^\infty d\omega'\, J(\omega') - \int_0^\infty d\omega'\, J(\omega')\omega'\Big]. \tag{S7.8}$$

The real part, Re$[\Gamma_\pm(\Omega, t)]$, also increases linearly with time t but remains independent of the temperature T owing to zero-point fluctuations. Consequently, at low temperatures the dissipation arises solely from these fundamental quantum fluctuations, resulting in more slowly narrowing corridors and a longer preservation of coherence.

7.4 Definition of coupling regime through the ratio of renormalisation energy and eigen energy gap

Following the ratio as defined

$$R \equiv \frac{E_{\text{ren}}}{\Omega} \qquad \text{where} \quad E_{\text{ren}} = \int_0^\infty d\omega \, \frac{J_U(\omega)}{\omega}, \quad \text{and} \quad \Omega = \sqrt{\epsilon^2 + \Delta^2}. \tag{S7.9}$$

Accordingly, the weak-coupling regime corresponds to $R \ll 1$, the strong-coupling regime to $R \gtrsim 1$, and the intermediate regime typically lies within $R \approx 0.1$–0.5. The renormalisation energy E_{ren} for the under-damped Brownian-motion spectral density is given by

$$\begin{aligned} E_{\text{ren}} &= \gamma\lambda^2 \int_0^\infty \frac{d\omega}{(\omega^2 - \omega_0^2)^2 + \gamma^2\omega^2} \\ &= \gamma\lambda^2 \cdot \frac{1}{2} \int_{-\infty}^\infty \frac{d\omega}{(\omega^2 - \omega_0^2)^2 + \gamma^2\omega^2} \\ &= \gamma\lambda^2 \cdot \mathcal{I}, \end{aligned} \tag{S7.10}$$

where $\mathcal{I}$ is evaluated using contour integration. This yields the renormalisation energy

$$E_{\text{ren}} = \frac{\pi\lambda^2}{2\omega_0^2}. \tag{S7.11}$$

However, we define the parameter λ to depend on g_k for the system operator $\hat{\sigma}_x$. For the system operator $\sum_{m=1,2} \hat{\sigma}_m^+ \hat{\sigma}_m^-$, we consider each site that depend on $g_k^{(m)}$. Accordingly, the regimes of weak, intermediate, and strong coupling can be specified approximately for both system operators, as summarised in Table XX.

Table 5: Regimes of coupling xxx.

Regimes	$\hat{\sigma}_x$	
	g_k [Δ]	R
weak	0.2	0.0056
intermediate	0.5	0.352
strong	1.0	1.405

* *

$\sum_{m=1,2} \hat{\sigma}_m^+ \hat{\sigma}_m^-$			
$g_k^{(1)}$ [Δ]	$R_1 = E_1/\Omega$	$g_k^{(2)}$ [Δ]	$R_2 = E_2/\Omega$
2.0	5.621 (strong)	0.1	0.014 (weak)
		0.5	0.352 (intermediate)
		1.0	1.405 (strong)
		4.0	22.483 (vary strong)

References

Blau, Samuel M., Doran I. G. Bennett, Christoph Kreisbeck, Gregory D. Scholes & Alán Aspuru-Guzik. 2018. Local protein solvation drives direct down-

conversion in phycobiliprotein pc645 via incoherent vibronic transport. *Proc. Natl. Acad. Sci. U.S.A.* 115(15). E3342–E3350. https://doi.org/10.1073/pnas.1800370115.

Feynman, Richard P., Jr. Vernon, Frank L. & Robert W. Hellwarth. 1957. Geometrical representation of the schrödinger equation for solving maser problems. *J. Appl. Phys.* 28(1). 49–52. https://doi.org/10.1063/1.1722572.

Kirchner, Stefan. 2010. Spin path integrals, berry phase, and the quantum phase transition in the sub-ohmic spin-boson model. *J. Low Temp. Phys.* 161. 282–298. https://doi.org/10.1007/s10909-010-0193-4.

Lambert, Neill, Tarun Raheja, Simon Cross, Paul Menczel, Shahnawaz Ahmed, Alexander Pitchford, Daniel Burgarth & Franco Nori. 2023. Qutip-bofin: A bosonic and fermionic numerical hierarchical-equations-of-motion library with applications in light-harvesting, quantum control, and single-molecule electronics. *Phys. Rev. Res.* 5. 013181. https://doi.org/10.1103/PhysRevResearch.5.013181.

Uthailiang, Teerapat, Ongart Suntijitrungruang, Purin Issarakul, Peera Pongkitiwanichakul & S. Boonchui. 2025. Investigation of quantum trajectories in photosynthetic light harvesting through a quantum stochastic approach. *Sci. Rep.* 15. 5220. https://doi.org/10.1038/s41598-025-89474-3.
